# In(III)-TMSBr-Catalyzed Cascade Reaction of Diarylalkynes with Acrylates for the Synthesis of Aryldihydronaphthalene Derivatives

**DOI:** 10.3390/molecules23040979

**Published:** 2018-04-23

**Authors:** Qiu-Chi Zhang, Wen-Wei Zhang, Liang Shen, Zhi-Liang Shen, Teck-Peng Loh

**Affiliations:** 1 Division of Chemistry and Biological Chemistry, School of Physical and Mathematical Sciences, Nanyang Technological University, Singapore 637371, Singapore; zhan0413@e.ntu.edu.sg (Q.-C.Z.); lshen003@e.ntu.edu.sg (L.S.); 2 BGI, BGI-Shenzhen, Shenzhen 518083, China; zhangww@genomics.cn; 3 Institute of Advanced Synthesis, School of Chemistry and Molecular Engineering, Jiangsu National Synergetic Innovation Center for Advanced Materials, Nanjing Tech University, Nanjing 210009, China; ias_zlshen@njtech.edu.cn

**Keywords:** indium, combined Lewis acid, catalysis, cascade reaction, aryldihydronaphthalene

## Abstract

A combined Lewis acid system comprising of two or more Lewis acids occasionally exhibits augmented catalytic activity in organic transformations which are otherwise unrealizable by either of the components exclusively. On the other hand, the efficient construction of multiple new C-C bonds and polycyclic structures in minimal steps remains a subject of great interest in both academia and industry. Herein we report an efficient method to assemble aryldihydronaphthalene derivatives via a cascade reaction of diarylalkynes with acrylates under the catalysis of a combined Lewis acid derived from In(III) salt and TMSBr.

## 1. Introduction

**Figure 1:**
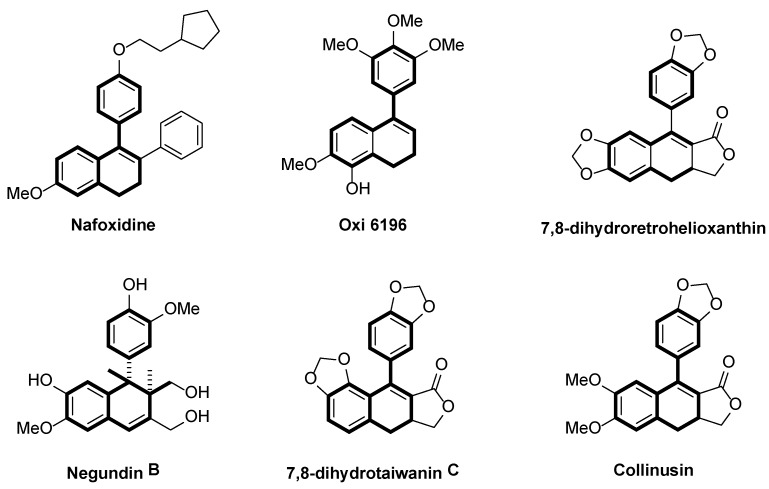
Representative natural products and bioactive compounds containing aryldihydronaphthalene structures [3,4,5,6,7].

In the context of green chemistry, efficient methods to forge multiple new C-C bonds and to introduce molecular complexity in minimal steps have been a long-standing and important goal in the development of modern organic chemistry [1,2]. In this regard, aryldihydronaphthalene derivatives are particularly attractive targets mainly due to their widespread occurrence in natural products and bioactive compounds (Figure 1) [3,4,5,6,7]. They are also employed as fluorescent ligands in biochemistry studies or building blocks towards the synthesis of several biologically-active cyclic molecules [8,9,10,11]. Consequently, their significant roles have driven the establishment of several synthetic methodologies for their preparation. Prominently, the intramolecular hydroarylation of 4-phenyl-1-butyne or its derivatives is one of the most versatile protocols for the construction of aryldihydronaphthalene derivatives [12,13,14,15]. Since the pioneering studies by Fujiwara et al. [16,17], numerous catalytic methods have been developed in this field in which a series of transition metals, Lewis and Bronsted acids have been found effective for catalyzing the hydroarylation [18,19,20,21,22,23,24,25,26,27,28,29,30,31,32,33,34,35,36,37,38,39]. More recently, Corey [24] and Pérez Sestelo and Martínez [38,39] have independently reported the formation of six-membered oxa- and carbocycles by an In(III)-catalyzed hydroarylation of acetylenic substrates.

**Scheme 1:**
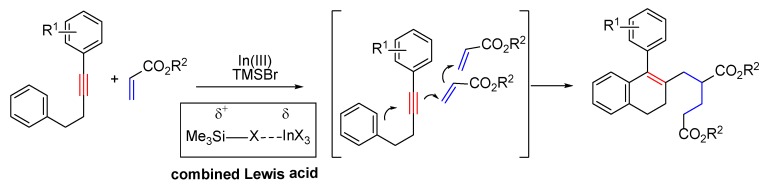
In(III)-TMSBr-catalysed cascade reaction of diarylalkynes with acrylates for the synthesis of dihydronaphthalene derivatives.

On the other hand, although indium(III) salts (InX_3_ (X = Cl, Br, I, OTf, NTf_2_, etc.)) have been widely applied as Lewis acids in organic synthesis [40,41,42], their relatively weak Lewis acidity nonetheless limit their applications. Unlike other group III elements, such as aluminum and boron, trimethylsilyl halide is often employed together with InX_3_ as a robust combined Lewis acid catalyst, as observed in various reactions developed by Baba [43,44], Lee [45], Corey [24,46], our group [47,48,49], and others. As a continuation of our research interest in the application of indium in organic synthesis [47,48,49,50,51], herein we wish to report the first example of the In(III)-TMSBr**-**catalysed cascade reaction of diarylalkynes with acrylates to access a series of dihydronaphthalene derivatives in a one-pot manner (Scheme 1).

## 2. Results

### 2.1. Preliminary Result of In(III)-TMSBr-Catalyzed Cascade Reaction of Diarylalkynes with Acrylates

**Scheme 2:**
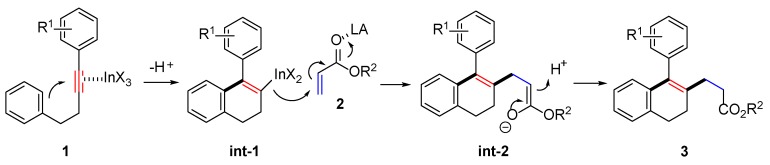
Proposed In(III)-TMSBr-catalysed cascade reaction of diarylalkynes with acrylates.

More recently, our group has demonstrated that the combined Lewis acid of In(III) salt and TMSBr could effectively activate acrylate component for the reaction with aryl alkynes [49]. Inspired by this activation mode, we envisaged a cascade reaction between diarylalkyne **1** and acrylate **2** to prepare aryldihydronaphthalene derivatives with this combined Lewis acid via the reaction sequence shown in Scheme 2. Initially, an intramolecular Friedel-Crafts type arylation reaction might occur, to give alkenyl indium species **int-1** which will then undergo nucleophilic attack on the activated acrylate **2** to give the dihydronaphthalene enolate **int-2**. A further enolate protonation will give the final 1,2-dihydronaphthalene derivative **3**.

**Scheme 3:**
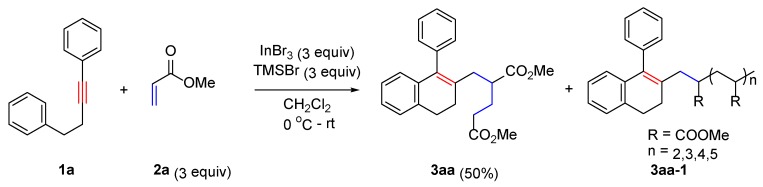
Initial results of In(III)-TMSBr-catalysed cascade reaction of but-1-yne-1,4-diyldibenzene (**1a**) with methyl acrylates (**2a**).

To begin with, the model reaction involving but-1-yne-1,4-diyldibenzene (**1a**) and methyl acrylate (**2a**) were investigated to probe the proposed cascade reaction. In the presence of InBr_3_ and TMSBr, a 1,2-dihydronaphthalene derivative **3aa** with two propionate motifs was obtained in moderate yield with trace amounts of intractable mixture of 1,2-dihydronaphthalene derivatives containing more propionate motifs (Scheme 3). Interestingly, no 1,2-dihydronaphthalene derivative with a single propionate motif in original proposal could be obtained.

### 2.2. Optimization of In(III)-TMSBr-Catalyzed Cascade Reaction of Diarylalkynes with Acrylates

We subsequently optimized the reaction conditions by using but-1-yne-1,4-diyldibenzene (**1a**) and methyl acrylate (**2a**) as model substrates. The results are summarized in Table 1. At the outset, it was found that both In(III) catalyst and TMSBr were indispensable for the efficient progress of this reaction, because the reaction could not take place in the absence of either of them (entries 2–3). Among the different indium catalysts studied (entries 1 and 4–8), In(tfacac)_3_ was found to exhibit the best catalytic activity to afford the desired product **3aa** with 61% yield (entry 7). Other common Lewis acid catalysts (e.g., AlBr_3_ and ZnCl_2_, entries 9 and 10) were also screened, which mostly resulted in no product formation. In addition, this reaction was found to proceed only in chlorinated solvents, such as CH_2_Cl_2_ or 1,2-dichloroethane, with the latter giving a higher yield of 70% (entry 7 vs. entry 11). In comparison, when TMSBr was replaced by TMSCl, the product yield eroded significantly to less than 5% (entry 12). An attempt to decrease the amount of TMSBr or In(tfacac)_3_ led to lower yields (entries 13–14). Finally, reducing the stoichiometry of methyl acrylate (**2a**) to only one equivalent resulted in a significantly decreased yield of **3aa**, and 1,2-dihydronaphthalene derivative with a single propionate motif remained absent (entry 15).

### 2.3. Substrate Scope of In(III)-TMSBr-Catalysed Cascade Reaction of Diarylalkynes with Acrylates

**Figure 2:**
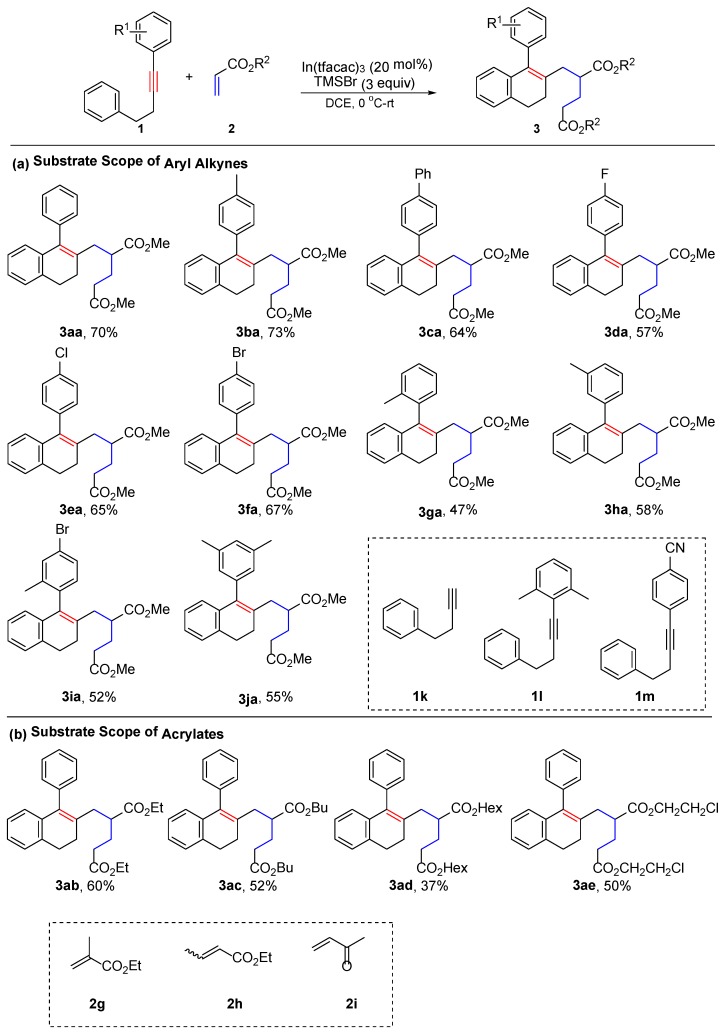
Substrate scope for the In(III)-TMSBr-catalysed cascade reaction of diarylalkyne with acrylates ^a,b^. ^a^ Unless otherwise noted, all reactions were performed with **1** (0.4 mmol), **2** (1.2 mmol), In(tfacac)_3_ (20 mol %), TMSBr (3 equiv), 0 °C-rt, N_2_. ^b^ Isolated yields.

With the optimized reaction conditions in hand (Table 1, entry 11), the generality of diarylalkyne substrate scope of this reaction with respect to methyl acrylate (**2a**) was investigated, and the results are listed in Figure 2a. Various substituted but-1-yne-1,4-diyldibenzene derivatives on the phenyl ring were well suited for this protocol, producing the corresponding product **3** in 47% to 73% yields (**3aa**-**ja**). Expectedly, substrates with *orth*-substituent on phenyl ring gave respective products in lower yields than those with *meta*- or *para*-substituents (**3ga** vs. **3ba** and **3ha**, **3fa** vs. **3ia**) and no cyclization product could be detected when **1l** was used under the same conditions. Additionally, the incompatibility of 4-phenyl-1-butyne (**1k**) with the current transformation emphasized the importance of the phenyl ring moiety for this reaction. Finally, a substrate with a strong aryl electron-withdrawing substituent (e.g., **1m**) was also unsuitable for this reaction.

With but-1-yne-1,4-diyldibenzene (**1a**) as the standard coupling partner, next, the scope of acrylate **2** in the present protocol was examined (Figure 2b). In addition to methyl acrylate (**3a**), acrylates carrying longer *O*-alkyl chains also reacted smoothly to give 1,2-dihydronaphthalene products **3ab-ad**, albeit in relatively low yields (37–60%). Aside from the alkyl chain, reactions of acrylates tethering the chloroethyl group proceeded well to give **3ae** in moderate yield (50%). However, the use of other olefinic substrates, such as ethyl methacrylate (**2g**), ethyl but-2-enoate (**2h**), and but-3-en-2-one (**2i**), could not provide any desired product.

## 3. Discussion

**Scheme 4:**
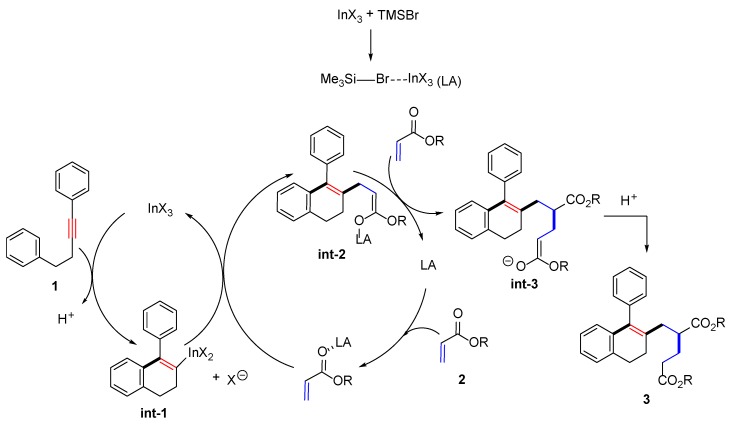
Plausible reaction mechanism of the In(tfacac)_3_-TMSBr-catalysed cascade reaction of diarylalkynes with acrylates.

On the basis of above experimental results and precedent literature reports [38,39,43,44,49], a plausible reaction pathway for this In(tfacac)_3_-TMSBr-catalyzed cascade reaction of diarylalkynes with acrylates was put forward in Scheme 4: In(tfacac)_3_ and TMSBr would first form a combined Lewis acid complex (LA) with heightened acidity than either of them solely; second, an intramolecular Friedel-Crafts type arylation reaction takes place to generate an alkenyl indium species **int-1**; this is followed by a nucleophilic attack of **int-1** onto activated acrylate **2**, giving rise to **int-2**, which subsequently attacks another molecule of acrylate to give **int-3**; finally, the **int-3** is quenched by the proton generated from the first step to furnish the final product **3**.

## 4. Materials and Methods 

### 4.1. General Information

Unless otherwise noted, all reagents and solvents were purchased from commercial sources and used as received. The TMSBr and InBr_3_ used was purchased from Sigma-Aldrich, Singapore. The In(tfacac)_3_ used was purchased from Strem Chemicals (Newburyport, MA, USA) and its purity is 99%. Thin layer chromatography (TLC) was used to monitor the reaction progress on Merck (Frankfurter Strasse, Darmstadt, Germany) 60 F254 precoated silica gel plates (0.2 mm thickness). TLC spots were visualized by UV-light irradiation on a Spectro line model ENF-24061/F (Spectroline, Westbury, NY, USA) at 254 nm. Anther visualization method was staining with a basic solution of potassium permanganate or acidic solution of ceric molybdate, followed by heating. Flash column chromatography was performed using Merck silica gel 60 (Frankfurter Strasse, Darmstadt, Germany) with analytical grade solvents as eluents. ^1^H-NMR, ^13^C-NMR, and 2D NMR spectra were recorded using Bruker Avance 400 MHz spectrometers (Bruker Corporation, Billerica, MA, USA). Corresponding chemical shifts are reported in ppm downfield relative to TMS and were referenced to the signal of chloroform-d (δ = 7.26, singlet). Multiplicities were given as: s = singlet, d = doublet, t = triplet, q = quartet, m = multiplet, brs = broad singlet, dd = doublet of doublets, and td = triplet of doublets. Values of coupling constants are reported as *J* in Hz.

### 4.2. General Procedure for In(III)-TMSBr-Catalysed Cascade Reaction of Diarylalkyne with Acrylates

A dry reaction tube was charged with aryl alkyne **1** (0.4 mmol), acrylate **2** (1.2 mmol), indium(III) trifluoroacetylacetonate (In(tfacac)_3_, 20 mol %, 0.08 mmol, 45.9 mg) and DCE (1 mL) under N_2_ atmosphere at 0 °C. Bromotrimethylsilane (TMSBr, 3 equiv, 1.2 mmol, 183.6 mg) was added and the reaction mixture was stirred at room temperature for 2 h. Upon completion of the reaction as indicated by TLC analysis, the residue was directly purified by flash column chromatography on silica gel (eluent: hexane/ethyl acetate 10:1) to afford the desired product **3**.

### 4.3. Product Characterization

*Dimethyl 2-((1-phenyl-3,4-dihydronaphthalen-2-yl)methyl)pentanedioate* (**3aa**). Colorless oil, 106.2 mg, 0.281 mmol, 70% yield. ^1^H-NMR (400 MHz, CDCl_3_): δ (ppm) 7.45–7.41 (m, 2H), 7.38–7.34 (m, 1H), 7.18–7.10 (m, 4H), 7.05–7.02 (m, 1H), 6.58 (d, *J* = 7.5 Hz, 1H), 3.67 (s, 3H), 3.65 (s, 3H), 2.91 (t, *J* = 7.7 Hz, 2H), 2.66–2.61 (m, 1H), 2.44–2.31 (m, 4H), 2.22–2.18 (m, 2H), 1.79–1.76 (m, 2H); ^13^C-NMR (100 MHz, CDCl_3_): δ (ppm) 175.4, 173.3, 139.2, 136.7, 136.3, 135.2, 134.3, 130.2, 128.4, 127.0, 126.9, 126.4, 126.2, 125.8, 51.6, 51.5, 43.5, 37.0, 31.6, 28.4, 27.4, 26.7.

*Dimethyl 2-((1-(p-tolyl)-3,4-dihydronaphthalen-2-yl)methyl)pentanedioate* (**3ba**). Colorless oil, 114.5 mg, 0.292 mmol, 73% yield. ^1^H-NMR (400 MHz, CDCl_3_): δ (ppm) 7.24–7.22 (m, 2H), 7.17–7.12 (m, 1H), 7.11–7.08 (m, 1H), 7.05–7.01 (m, 3H), 6.60 (d, *J* = 7.0 Hz, 1H), 3.67 (s, 3H), 3.65 (s, 3H), 2.89 (t, *J* = 7.8 Hz, 2H), 2.66–2.62 (m, 1H), 2.44 (s, 3H), 2.43–2.35 (m, 4H), 2.22–2.18 (m, 2H), 1.79–1.74 (m, 2H); ^13^C-NMR (100 MHz, CDCl_3_): δ (ppm) 175.5, 173.3, 136.8, 136.4, 136.2, 136.0, 135.2, 134.2, 130.1, 129.1, 127.0, 126.3, 126.2, 125.9, 51.6, 51.5, 43.4, 37.0, 31.6, 28.4, 27.4, 26.6, 21.2.

*Dimethyl 2-((1-([1,1′-biphenyl]-4-yl)-3,4-dihydronaphthalen-2-yl)methyl)pentanedioate* (**3ca**). Colorless oil, 116.3 mg, 0.256 mmol, 64% yield. ^1^H-NMR (400 MHz, CDCl_3_): δ (ppm) 7.71–7.66 (m, 4H), 7.51–7.47 (m, 2H), 7.44–7.37 (m, 2H), 7, 23 (brs, 2H), 7.15–7.11 (m, 1H), 7.08–7.04 (m, 1H), 6.67 (d, *J* = 7.6 Hz, 1H), 3.66 (s, 3H), 3.65 (s, 3H), 2.94–2.90 (m, 2H), 2.70–2.66 (m, 1H), 2.50–2.41 (m, 4H), 2.25–2.19 (m, 2H), 1.83–1.77 (m, 2H); ^13^C-NMR (100 MHz, CDCl_3_): δ (ppm) 175.5, 173.3, 140.9, 139.6, 138.2, 136.7, 135.9, 135.2, 134.5, 130.7 (×2), 128.8, 127.3, 127.1 (×2), 126.4, 126.2, 125.9, 51.7, 51.6, 43.5, 37.0, 31.6, 28.4, 27.4, 26.6.

*Dimethyl 2-((1-(4-fluorophenyl)-3,4-dihydronaphthalen-2-yl)methyl)pentanedioate* (**3da**). Colorless oil, 90.3 mg, 0.228 mmol, 57% yield. ^1^H-NMR (400 MHz, CDCl_3_): δ (ppm) 7.18–7.16 (m, 1H), 7.14–7.10 (m, 5H), 7.07–7.03 (m, 1H), 6.56–6.54 (d, *J* = 7.5 Hz, 1H), 3.68 (s, 3H), 3.65 (s, 3H), 2.90 (t, *J* = 7.9 Hz, 2H), 2.66–2.61 (m, 1H), 2.44–2.29 (m, 4H), 2.24–2.19 (m, 2H), 1.80–1.73 (m, 2H); ^13^C-NMR (100 MHz, CDCl_3_): δ (ppm) 175.3, 173.3, 161.9 (d, *J*_C-F_ = 243.7 Hz), 136.5, 135.3, 135.2, 134.9 (d, *J*_C-F_ = 4.0 Hz), 134.8, 131.8 (d, *J*_C-F_ = 7.8 Hz), 127.1, 126.5, 126.2, 125.7, 115.3 (d, *J*_C-F_ = 21.1 Hz), 51.7, 51.6, 43.4, 37.0, 31.5, 28.3, 27.4, 26.7.

*Dimethyl 2-((1-(4-chlorophenyl)-3,4-dihydronaphthalen-2-yl)methyl)pentanedioate* (**3ea**). Colorless oil, 107.1 mg, 0.26 mmol, 65% yield. ^1^H-NMR (400 MHz, CDCl_3_): δ (ppm) 7.61–7.59 (m, 2H), 7.22–7.14 (m, 3H), 7.10–7.06 (m, 2H), 6.58 (d, *J* = 7.6 Hz, 1H), 3.72 (s, 3H), 3.69 (s, 3H), 2.93 (t, *J* = 7.8 Hz, 2H), 2.71–2.67 (m, 1H), 2.48–2.41 (m, 4H), 2.37–2.32 (m, 2H), 1.83–1.77 (m, 2H); ^13^C-NMR (100 MHz, CDCl_3_): δ (ppm) 175.3, 173.2, 138.1, 136.2, 135.2, 135.1, 134.8, 132.0, 131.6, 127.1, 126.6, 126.3, 125.7, 121.0, 51.7, 51.6, 43.3, 37.0, 31.5, 28.3, 27.3, 26.7.

*Dimethyl 2-((1-(4-bromophenyl)-3,4-dihydronaphthalen-2-yl)methyl)pentanedioate* (**3fa**). Colorless oil, 122.2 mg, 0.268 mmol, 67% yield. ^1^H-NMR (400 MHz, CDCl_3_): δ (ppm) 7.41–7.39 (m, 2H), 7.17–7.02 (m, 5H), 6.54 (d, *J* = 7.6 Hz, 1H), 3.67 (s, 3H), 3.65 (s, 3H), 2.89 (t, *J* = 7.9 Hz, 2H), 2.68–2.61 (m, 1H), 2.43–2.26 (m, 4H), 2.24–2.19 (m, 2H), 1.79–1.75 (m, 2H); ^13^C-NMR (100 MHz, CDCl_3_): δ (ppm) 175.3, 173.2, 137.6, 136.3, 135.1, 134.9, 132.9, 131.7, 128.7 (×2), 127.1, 126.6, 126.3, 125.7, 51.7, 51.6, 43.3, 37.0, 31.5, 28.3, 27.3, 26.7.

*Dimethyl 2-((1-(o-tolyl)-3,4-dihydronaphthalen-2-yl)methyl)pentanedioate* (**3ga**). Colorless oil, 73.7 mg, 0.188 mmol, 47% yield. ^1^H-NMR (400 MHz, CDCl_3_): δ (ppm) 7.28–7.22 (m, 3H), 7.18–7.17 (m, 1H), 7.12–7.08 (m, 2H), 7.03–7.00 (m, 1H), 6.49 (d, *J* = 7.4 Hz, 1H), 3.66 (s, 3H), 3.65 (s, 3H), 2.92–2.90 (m, 2H), 2.64–2.61 (m, 1H), 2.46–2.29 (m, 4H), 2.19–2.15 (m, 2H), 2.05 (s, 3H), 1.79–1.72 (m, 2H); ^13^C-NMR (100 MHz, CDCl_3_): δ (ppm) 175.7, 173.3, 138.3, 136.8, 135.8, 135.4, 135.1, 134.2, 130.6, 130.4, 130.1, 127.3, 127.0, 126.4, 125.8, 125.1, 51.6, 51.5, 43.3, 36.8, 31.5, 28.4, 27.1, 26.5, 19.3. 

*Dimethyl 2-((1-(m-tolyl)-3,4-dihydronaphthalen-2-yl)methyl)pentanedioate* (**3ha**). Colorless oil, 90.9 mg, 0.232 mmol, 58% yield. ^1^H-NMR (400 MHz, CDCl_3_): δ (ppm)7.32–7.30 (m, 1H), 7.17–7.15 (m, 2H), 7.12–7.08 (m, 1H), 7.05–7.01 (m, 1H), 6.97–6.93 (m, 2H), 6.59 (d, *J* = 7.5 Hz, 1H), 3.66 (s, 3H), 3,65 (s, 3H), 2.91–2.87 (m, 2H), 2.65–2.62 (m, 1H), 2.43–2.34 (m, 4H), 2.39 (s, 3H), 2.22–2.17 (m, 2H), 1.79–1.76 (m, 2H); ^13^C-NMR (100 MHz, CDCl_3_): δ (ppm) 175.6, 173.3, 139.0, 136.7, 136.3, 135.1, 134.1, 130.8, 128.2, 127.6 (×2), 127.2, 127.0, 126.3, 126.2, 125.9, 51.6, 51.5, 43.4, 36.9, 31.6, 28.4, 27.3, 26.6, 21.5.

*Dimethyl 2-((1-(4-bromo-2-methylphenyl)-3,4-dihydronaphthalen-2-yl)methyl)pentanedioate* (**3ia**). Colorless oil, 97.8 mg, 0.208 mmol, 52% yield. ^1^H-NMR (400 MHz, CDCl_3_): δ (ppm) 7.44 (s, 1H), 7.38 (d, *J* = 8.1 Hz, 1H), 7.17 (d, *J* = 7.1 Hz, 1H), 7.13–7. 10 (m, 1H), 7.04–7.00 (m, 1H), 6.97 (d, *J* = 8.1 Hz, 1H), 6.45 (d, *J* = 7.4 Hz, 1H), 3.67 (s, 3H), 3.66 (s, 3H), 2.92–2.88 (m, 2H), 2.64–2.62 (m, 1H), 2.46–2.34 (m, 4H), 2.22–2.11 (m, 2H), 2.03 (s, 3H), 1.80–1.70 (m, 2H); ^13^C-NMR (100 MHz, CDCl_3_): δ (ppm) 175.5, 173.2, 139.4, 137.3, 135.3, 135.1, 134.8, 134.3, 133.0, 132.1, 129.0, 127.2, 126.6, 126.4, 124.9, 121.1, 51.7, 51.6, 43.2, 36.9, 31.5, 28.3 (×2), 27.0, 26.5.

*Dimethyl 2-((1-(3,5-dimethylphenyl)-3,4-dihydronaphthalen-2-yl)methyl)pentanedioate* (**3ja**). Colorless oil, 89.4 mg, 0.220 mmol, 55% yield. ^1^H-NMR (400 MHz, CDCl_3_): δ (ppm) 7.17–7.15 (m, 1H), 7.12–7.08 (m, 1H), 7.06–7.02 (m, 1H), 6.98 (s, 1H), 6.78 (s, 1H), 6.73 (s, 1H), 6.62 (d, *J* = 7.5 Hz, 1H), 3.67 (s, 3H), 3.66 (s, 3H), 2.91–2.86 (m, 2H), 2.66–2.61 (m, 1H), 2.44–2.37 (m, 4H), 2.35 (s, 6H), 2.24–2.14 (m, 2H), 1.81–1.75 (m, 2H); ^13^C-NMR (100 MHz, CDCl_3_): δ (ppm) 175.7, 173.3, 139.0, 137.7, 136.8, 136.4, 135.1, 133.9, 128.4, 127.9, 126.9, 126.2, 126.1, 125.9, 51.6, 51.5, 43.5, 37.0, 31.6, 28.4, 27.3, 26.6, 21.3.

*Diethyl 2-((1-phenyl-3,4-dihydronaphthalen-2-yl)methyl)pentanedioate* (**3ab**). Colorless oil, 97.5 mg, 0.240 mmol, 60% yield. ^1^H-NMR (400 MHz, CDCl_3_): δ (ppm) 7.44–7.40 (m, 2H), 7.37–7.33 (m 1H), 7.18–7.16 (m, 3H), 7.12–7.09 (m, 1H), 7.05–7.01 (m, 1H), 6.58 (d, *J* = 7.6 Hz, 1H), 4.17–4.08 (m, 4H), 2.90 (t, *J* = 8.3 Hz, 1H), 2.65–2.58 (m, 1H), 2.43–2.30 (m, 4H), 2.21–2.14 (m, 2H), 1.79–1.74 (m, 2H), 1.29–1.27 (m, 6H); ^13^C-NMR (100 MHz, CDCl_3_): δ (ppm) 175.1, 172.9, 139.2, 136.7, 136.2, 135.2, 134.5, 130.3, 128.4, 127.0, 126.8, 126.3, 126.2, 125.8, 60.4, 60.3, 43.5, 37.0, 31.8, 28.4, 27.4, 26.8, 14.2.

*Dibutyl 2-((1-phenyl-3,4-dihydronaphthalen-2-yl)methyl)pentanedioate*(**3ac**). Colorless oil, 96.2 mg, 0.208 mmol, 52% yield. ^1^H-NMR (400 MHz, CDCl_3_): δ (ppm) 7.44–7.40 (m, 2H), 7.37–7.33 (m, 1H), 7.17–7.09 (m, 4H), 7.05–7.01 (m, 1H), 6.58 (d, *J* = 7.0 Hz, 1H), 4.07–4.03 (m, 4H), 2.91 (t, *J* = 8.1 Hz, 2H), 2.66–2.57 (m, 1H), 2.47–2.30 (m, 4H), 2.21–2.12 (m, 2H), 1.81–1.73 (m, 2H), 1.65–1.54 (m, 4H), 1.42–1.29 (m, 4H), 0.97–0.89 (m, 6H); ^13^C-NMR (100 MHz, CDCl_3_): δ (ppm) 175.1, 173.0, 139.2, 136.7, 136.2, 135.1, 134.4, 130.3, 128.4, 127.0, 126.8, 126.3, 126.2, 125.8, 64.4, 64.3, 43.5, 37.0, 31.9, 30.7, 30.6, 28.4, 27.4, 26.8, 19.1 (×2), 13.7, 13.6.

*Dihexyl 2-((1-phenyl-3,4-dihydronaphthalen-2-yl)methyl)pentanedioate*(**3ad**). Colorless oil, 66.1 mg, 0.148 mmol, 37% yield. ^1^H-NMR (400 MHz, CDCl_3_): δ (ppm) 7.44–7.40 (m, 2H), 7.37–7.35 (m, 1H), 7.17–7.16 (m, 3H), 7.12–7.08 (m, 1H), 7.05–7.01 (m, 1H), 6.57 (d, *J* = 7.5 Hz, 1H), 4.09–4.02 (m, 4H), 2.92–2.88 (m, 2H), 2.64–2.62 (m, 1H), 2.45–2.32 (m, 4H), 2.22–2.16 (m, 2H), 1.79–1.71 (m, 2H), 1.65–1.56 (m, 6H), 1.32–1.28 (m, 10H), 0.93–0.87 (m, 6H); ^13^C-NMR (100 MHz, CDCl_3_): δ (ppm) 175.1, 173.0, 139.2, 136.7, 136.2, 135.1, 134.4, 130.3, 128.4, 127.0, 126.8, 126.3, 126.2, 125.8, 64.7, 64.6, 43.5, 37.0, 31.8, 31.4, 31.3, 28.6, 28.5, 28.4, 27.4, 26.7, 25.6 (×2), 22.5, 22.4, 14.0 (×2).

*Bis(2-chloroethyl) 2-((1-phenyl-3,4-dihydronaphthalen-2-yl)methyl)pentanedioate* (**3ae**). Colorless oil, 89.2 mg, 0.200 mmol, 50% yield. ^1^H-NMR (400 MHz, CDCl_3_): δ (ppm) 7.45–7.43 (m, 2H), 7.38–7.36 (m, 1H), 7.18–7.10 (m, 4H), 7.06–7.02 (m, 1H), 6.58 (d, *J* = 7.6 Hz, 1H), 4.35–4.29 (m, 4H), 3.70–3.63 (m, 4H), 2.94–2.89 (m, 2H), 2.73–2.67 (m, 1H), 2.50–2.33 (m, 4H), 2.31–2.26 (m, 2H), 1.84–1.79 (m, 2H); ^13^C-NMR (100 MHz, CDCl_3_): δ (ppm) 174.6, 172.4, 139.1, 136.6, 136.4, 135.1, 134.0, 130.2, 128.4, 127.0, 126.9, 126.4, 126.2, 125.9, 64.0, 63.9, 43.2, 41.5, 41.4, 36.9, 31.5, 28.4, 27.4, 26.4.

Product characterization data, and ^1^H- and ^13^C-NMR spectra are available from supplementary material.

## 5. Conclusions

In a nutshell, we described an efficient method to assemble aryldihydronaphthalene derivatives via the cascade reaction of diarylalkynes with acrylates employing the catalysis of a combined Lewis acid system formed from In(III) salt and TMSBr. Both indium(III) and TMSBr are indispensable for the efficient progress of the reaction. In most cases, the reaction proceeded efficiently to afford the corresponding aryldihydronaphthalene derivatives in moderate to good yields. With reference to current experimental observations and literature reports, a possible mechanistic pathway for this reaction was also provided.

## Figures and Tables

**Table 1:**
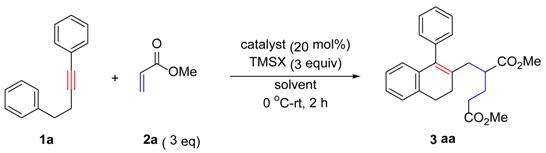
The In(III)-TMSBr-catalyzed cascade reaction of diarylalkyne **1a** with methyl acrylate (**2a**) ^a^.

Entry	Catalyst	Solvent	TMSX	Yield (%) ^b^
1	InBr_3_	CH_2_Cl_2_	TMSBr	50
2	InBr_3_	CH_2_Cl_2_	-	0
3	-	CH_2_Cl_2_	TMSBr	0 ^c^
4	InCl_3_	CH_2_Cl_2_	TMSBr	14
5	InI_3_	CH_2_Cl_2_	TMSBr	47
6	In(OTf)_3_	CH_2_Cl_2_	TMSBr	37
7	In(tfacac)_3_	CH_2_Cl_2_	TMSBr	61
8	In(acac)_3_	CH_2_Cl_2_	TMSBr	0
9	AlBr_3_	CH_2_Cl_2_	TMSBr	0
10	ZnCl_2_	CH_2_Cl_2_	TMSBr	0
11	In(tfacac)_3_	ClCH_2_CH_2_Cl	TMSBr	70
12	In(tfacac)_3_	ClCH_2_CH_2_Cl	TMSCl	<5
13	In(tfacac)_3_	ClCH_2_CH_2_Cl	TMSBr	60 ^c^
14	In(tfacac)_3_	ClCH_2_CH_2_Cl	TMSBr	57 ^d^
15	In(tfacac)_3_	ClCH_2_CH_2_Cl	TMSBr	33 ^e^

^a^ Unless otherwise noted, all reactions were performed with **1a** (0.4 mmol), **2a** (1.2 mmol), catalyst (20 mol %), TMSX (3 equiv), 0 °C-rt, 2 h, N_2_. ^b^ Isolated yields. ^c^ 2.0 equiv of TMSBr was added. ^d^ 10 mol % of In(tfacac)_3_ was added. ^e^ 1.0 equiv of **2a** was added.

## Supplementary Materials

Supplementary materials are available online: general experimental procedures, product characterization data, and ^1^H- and ^13^C-NMR spectra.
