# Supplementary material for: In(III)-TMSBr-Catalyzed Cascade Reaction of Diarylalkynes with Acrylates for the Synthesis of Aryldihydronaphthalene Derivatives
Source: Molecules. 2018 Apr 23;23(4):979. doi: 10.3390/molecules23040979 (PMC6017889; doi:10.3390/molecules23040979)
Supplement: Supplementary file 1 [file molecules-23-00979-s001.pdf]

Supporting Information

In(III)-TMSBr-Catalyzed Cascade Reaction of Diarylalkynes with Acrylates for The Synthesis of Aryldihydronaphthalene Derivatives

Qiu-Chi Zhang [3,†], Wen-Wei Zhang [2,†], Liang Shen [3], Zhi-Liang Shen [1] and Teck-Peng Loh [1,3,*]

[1] Institute of Advanced Synthesis, School of Chemistry and Molecular Engineering, Jiangsu National Synergetic Innovation Center for Advanced Materials, Nanjing Tech University, Nanjing, Jiangsu, P. R. China 210009

[2] BGI, BGI-Shenzhen, Shenzhen, China 518083

[3] Division of Chemistry and Biological Chemistry, School of Physical and Mathematical Sciences, Nanyang Technological University, Singapore 637371

† These authors contributed equally to this work and should be considered joint first authors.

Table of Contents

1. General Information S2

2. Experimental Sections
2.1 General procedure of In(III)-TMSBr-catalyzed Cascade Reaction of **1** with **2** S3
2.2 Product Characterization and Spectral Data S3

3. NMR Spectra S8

1. General Information

Unless otherwise noted, all reagents and solvents were purchased from the commercial sources and used as received.The TMSBr and $InBr_3$ used was purchased from Sigma-Aldrich.

Thin layer chromatography (TLC) was used to monitor the reaction on Merck 60 F254 precoated silica gel plate (0.2 mm thickness). TLC spots were visualized by UV-light irradiation on Spectroline Model ENF-24061/F 254 nm. Other visualization method was staining with a basic solution ofpotassium permanganate or acidic solution of ceric molybdate, followed by heating.

Flash column chromatography was performed using Merck silica gel 60 with analytical grade solvents as eluents.

^{1}H NMR, ^{13}C NMR and 2D NMR spectra were recorded using BrukerAvance 400 MHz spectrometers. Corresponding chemical shifts are reported in ppm downfield relative to TMS and were referenced to the signal of chloroform-d (δ=7.26, singlet). Multiplicities were given as: s = singlet, d = doublet, t = triplet, q = quartet, m = multiplet, brs = broad singlet, dd=doublet of doublets, td= triplet of doublets. Values of coupling constant are reported as *J* in Hz.

HRMS spectra were recorded on a Waters Q–TofPermier Spectrometer.

2. Experimental Sections

2.1 General procedure of In(III)-TMSBr-catalyzed Cascade Reaction of **1** with **2**

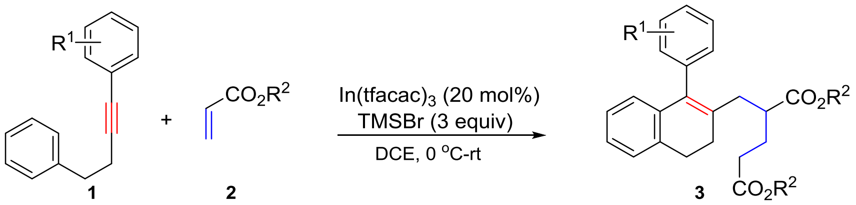

A dry reaction tube was charged with aryl alkyne **1** (0.4 mmol), acrylate **2** (1.2 mmol), indium (III) trifluoroacetylacetonate (In(tfacac)$_3$, 20 mol%, 0.08 mmol, 45.9 mg) and DCE (2 mL) under N_2 atmosphere at 0 °C. Bromotrimethylsilane (TMSBr, 3 equiv, 1.2 mmol, 183.6 mg) was added and the reaction mixture was stirred at room temperature for 2 h. Upon completion of the reaction as indicated by TLC analysis, the residue was directly purified by flash column chromatography on silica gel (eluent: hexane/ethyl acetate 10:1) to afford the desired product **3**.

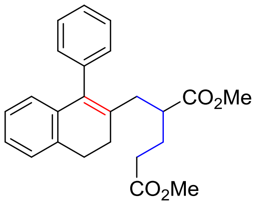

Dimethyl 2-((1-phenyl-3,4-dihydronaphthalen-2-yl)methyl) pentanedioate (3aa)

Colorless oil, 106.2 mg, 0.281 mmol, 70% yield.

^{1}H NMR (400 MHz, $CDCl_3$): δ (ppm) 7.45 - 7.41 (m, 2H), 7.38 - 7.34 (m, 1H), 7.18 - 7.10 (m, 4H), 7.05 -7.02 (m, 1H), 6.58 (d, J = 7.5 Hz, 1H), 3.67 (s, 3H), 3.65 (s, 3H), 2.91 (t, J = 7.7 Hz, 2H), 2.66 - 2.61 (m, 1H), 2.44 - 2.31 (m, 4H), 2.22 - 2.18 (m, 2H), 1.79 - 1.76 (m, 2H); **^{13}C NMR** (100 MHz, $CDCl_3$): δ (ppm) 175.4, 173.3, 139.2, 136.7, 136.3, 135.2, 134.3, 130.2, 128.4, 127.0, 126.9, 126.4, 126.2, 125.8, 51.6, 51.5, 43.5, 37.0, 31.6, 28.4, 27.4, 26.7.

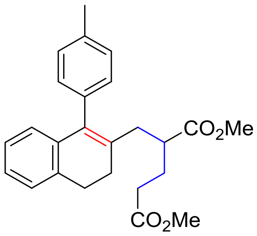

Dimethyl 2-((1-(*p*-tolyl)-3,4-dihydronaphthalen-2-yl)methyl) pentanedioate (3ba)

Colorless oil, 114.5 mg, 0.292 mmol, 73% yield.

^{1}H NMR (400 MHz, $CDCl_3$): δ (ppm) 7.24 - 7.22 (m, 2H), 7.17 - 7.12 (m, 1H), 7.11 - 7.08 (m, 1H), 7.05 - 7.01 (m, 3H), 6.60 (d, J = 7.0 Hz, 1H), 3.67 (s, 3H), 3.65 (s, 3H), 2.89 (t, J = 7.8 Hz, 2H), 2.66 -2.62 (m, 1H), 2.44 (s, 3H), 2.43 - 2.35 (m, 4H), 2.22 - 2.18 (m, 2H), 1.79 - 1.74 (m, 2H); **^{13}C NMR** (100 MHz, $CDCl_3$): δ (ppm) 175.5,

173.3, 136.8, 136.4, 136.2, 136.0, 135.2, 134.2, 130.1, 129.1, 127.0, 126.3, 126.2, 125.9, 51.6, 51.5, 43.4, 37.0, 31.6, 28.4, 27.4, 26.6, 21.2.

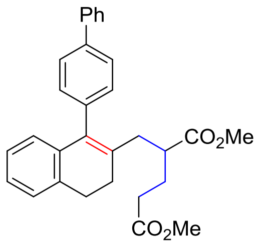

Dimethyl 2-((1-([1,1'-biphenyl]-4-yl)-3,4-dihydronaphthalen-2-yl) methyl) pentanedioate (3ca)

Colorless oil, 116.3 mg, 0.256 mmol, 64% yield.

^{1}H NMR (400 MHz, $CDCl_3$): δ (ppm) 7.71 - 7.66 (m, 4H), 7.51 - 7.47 (m, 2H), 7.44 - 7.37 (m, 2H), 7, 23 (br, 2H), 7.15 - 7.11 (m, 1H), 7.08 - 7.04 (m, 1H), 6.67 (d, J = 7.6 Hz, 1H), 3.66 (s. 3H). 3.65 (s, 3H), 2.94 - 2.90 (m, 2H), 2.70 - 2.66 (m, 1H), 2.50 - 2.41 (m, 4H), 2.25 - 2.19 (m, 2H), 1.83 - 1.77 (m, 2H); **^{13}C NMR** (100 MHz, $CDCl_3$): δ (ppm) 175.5, 173.3, 140.9, 139.6, 138.2, 136.7, 135.9, 135.2, 134.5, 130.7×2, 128.8, 127.3, 127.1×2, 126.4, 126.2, 125.9, 51.7, 51.6, 43.5, 37.0, 31.6, 28.4, 27.4, 26.6.

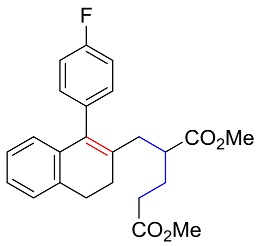

Dimethyl 2-((1-(4-fluorophenyl)-3,4-dihydronaphthalen-2-yl) methyl) pentanedioate (3da)

Colorless oil, 90.3 mg, 0.228 mmol, 57% yield.

^{1}H NMR (400 MHz, $CDCl_3$): δ (ppm) 7.18 - 7.16 (m, 1H), 7.14 - 7.10 (m, 5H), 7.07 - 7.03 (m, 1H), 6.56 - 6.54 (d, J = 7.5 Hz, 1H), 3.68 (s, 3H), 3.65 (s, 3H), 2.90 (t, J = 7.9 Hz, 2H), 2.66 - 2.61 (m, 1H), 2.44 - 2.29 (m, 4H), 2.24 - 2.19 (m, 2H), 1.80 - 1.73 (m, 2H); **^{13}C NMR** (100 MHz, $CDCl_3$): δ (ppm) 175.3, 173.3, 161.9 (d, J_{C-F} = 243.7 Hz), 136.5, 135.3, 135.2, 134.9 (d, J_{C-F} = 4.0 Hz), 134.8, 131.8 (d, J_{C-F} = 7.8 Hz), 127.1, 126.5, 126.2, 125.7, 115.3 (d, J_{C-F} = 21.1 Hz), 51.7, 51.6, 43.4, 37.0, 31.5, 28.3, 27.4, 26.7.

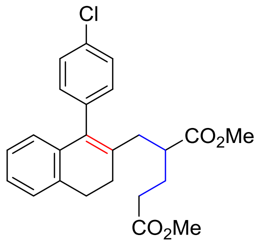

Dimethyl 2-((1-(4-chlorophenyl)-3,4-dihydronaphthalen-2-yl) methyl)pentanedioate (3ea)

Colorless oil, 107.1 mg, 0.260mmol, 65% yield.

^{1}H NMR (400 MHz, $CDCl_3$): δ (ppm) 7.61 - 7.59 (m, 2H), 7.22 - 7.14 (m, 3H), 7.10 - 7.06 (m, 2H), 6.58 (d, J = 7.6 Hz, 1H), 3.72 (s, 3H), 3.69 (s, 3H), 2.93 (t, J = 7.8 Hz, 2H), 2.71 - 2.67 (m, 1H), 2.48 - 2.41 (m, 4H), 2.37 - 2.32 (m, 2H), 1.83 - 1.77 (m, 2H); **^{13}C NMR** (100 MHz, $CDCl_3$): δ (ppm) 175.3, 173.2, 138.1, 136.2, 135.2, 135.1, 134.8, 132.0, 131.6, 127.1, 126.6, 126.3, 125.7, 121.0, 51.7, 51.6, 43.3, 37.0, 31.5, 28.3, 27.3, 26.7.

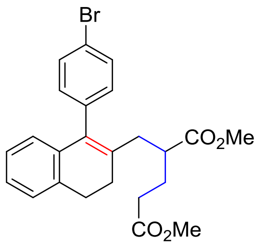

Dimethyl 2-((1-(4-bromophenyl)-3,4-dihydronaphthalen-2-yl) methyl)pentanedioate (3fa)

Colorless oil, 122.2 mg, 0.268 mmol, 67% yield.

^{1}H NMR (400 MHz, $CDCl_3$): δ (ppm) 7.41 - 7.39 (m, 2H), 7.17 - 7.02 (m, 5H), 6.54 (d, *J* = 7.6 Hz, 1H), 3.67 (s, 3H), 3.65 (s, 3H), 2.89 (t, *J* = 7.9 Hz, 2H), 2.68 - 2.61 (m, 1H), 2.43 - 2.26 (m, 4H), 2.24 - 2.19 (m, 2H), 1.79 - 1.75 (m, 2H); **^{13}C NMR** (100 MHz, $CDCl_3$): δ (ppm) 175.3, 173.2, 137.6, 136.3, 135.1, 134.9, 132.9, 131.7, 128.7×2, 127.1, 126.6, 126.3, 125.7, 51.7, 51.6, 43.3, 37.0, 31.5, 28.3, 27.3, 26.7.

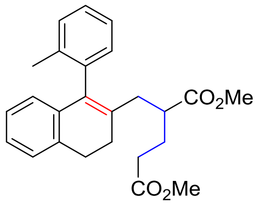

Dimethyl 2-((1-(*o*-tolyl)-3,4-dihydronaphthalen-2-yl)methyl) pentanedioate (3ga)

Colorless oil, 73.7 mg, 0.188 mmol, 47% yield.

^{1}H NMR (400 MHz, $CDCl_3$): δ (ppm) 7.28 - 7.22 (m, 3H), 7.18 - 7.17 (m, 1H), 7.12 - 7.08 (m, 2H), 7.03 - 7.00 (m, 1H), 6.49 (d, *J* = 7.4 Hz, 1H), 3.66 (s, 3H), 3.65 (s, 3H), 2.92 - 2.90 (m, 2H), 2.64 - 2.61 (m, 1H), 2.46 - 2.29 (m, 4H), 2.19 - 2.15 (m, 2H), 2.05 (s, 3H), 1.79 - 1.72 (m, 2H); **^{13}C NMR**(100 MHz, $CDCl_3$): δ (ppm) 175.7, 173.3, 138.3, 136.8, 135.8, 135.4, 135.1, 134.2, 130.6, 130.4, 130.1, 127.3, 127.0, 126.4, 125.8, 125.1, 51.6, 51.5, 43.3, 36.8, 31.5, 28.4, 27.1, 26.5, 19.3.

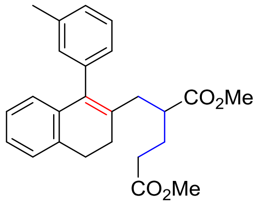

Dimethyl 2-((1-(*m*-tolyl)-3,4-dihydronaphthalen-2-yl)methyl) pentanedioate (3ha)

Colorless oil, 90.9 mg, 0.232 mmol, 58% yield.

^{1}H NMR (400 MHz, $CDCl_3$): δ (ppm)7.32 - 7.30 (m, 1H), 7.17 - 7.15 (m, 2H), 7.12 - 7.08 (m, 1H), 7.05 - 7.01 (m, 1H), 6.97 - 6.93 (m, 2H), 6.59 (d, *J* = 7.5 Hz, 1H), 3.66 (s, 3H), 3,65 (s, 3H), 2.91 - 2.87 (m, 2H), 2.65 - 2.62 (m, 1H), 2.43 - 2.34 (m, 4H), 2.39 (s, 3H), 2.22 - 2.17 (m, 2H), 1.79 - 1.76 (m, 2H); **^{13}C NMR** (100 MHz, $CDCl_3$): δ (ppm) 175.6, 173.3, 139.0, 136.7, 136.3, 135.1, 134.1, 130.8, 128.2, 127.6×2, 127.2, 127.0, 126.3, 126.2, 125.9, 51.6, 51.5, 43.4, 36.9, 31.6, 28.4, 27.3, 26.6, 21.5.

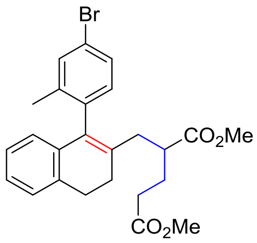

Dimethyl 2-((1-(4-bromo-2-methylphenyl)-3,4-dihydronaphthalen -2-yl) methyl)pentanedioate (3ia)

Colorless oil, 97.8 mg, 0.208 mmol, 52% yield.

^{1}H NMR (400 MHz, $CDCl_3$): δ (ppm) 7.44 (s, 1H), 7.38 (d, J = 8.1 Hz, 1H), 7.17 (d, J = 7.1 Hz, 1H), 7.13 - 7. 10 (m, 1H), 7.04 - 7.00 (m, 1H), 6.97 (d, J = 8.1 Hz, 1H), 6.45 (d, J = 7.4 Hz, 1H), 3.67 (s, 3H), 3.66 (s, 3H), 2.92 - 2.88 (m, 2H), 2.64 - 2.62 (m, 1H), 2.46 - 2.34 (m, 4H), 2.22 - 2.11 (m, 2H), 2.03 (s, 3H), 1.80 - 1.70 (m, 2H); **^{13}C NMR** (100 MHz, $CDCl_3$): δ (ppm) 175.5, 173.2, 139.4, 137.3, 135.3, 135.1, 134.8, 134.3, 133.0, 132.1, 129.0, 127.2, 126.6, 126.4, 124.9, 121.1, 51.7, 51.6, 43.2, 36.9, 31.5, 28.3×2, 27.0, 26.5.

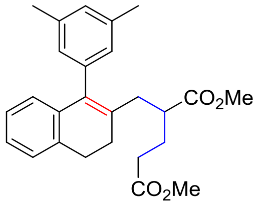

Dimethyl 2-((1-(3,5-dimethylphenyl)-3,4-dihydronaphthalen -2-yl)methyl) pentanedioate (3ja)

Colorless oil, 89.4 mg, 0.220 mmol, 55% yield.

^{1}H NMR (400 MHz, $CDCl_3$): δ (ppm) 7.17 - 7.15 (m, 1H), 7. 12 - 7.08 (m, 1H), 7.06 - 7.02 (m, 1H), 6.98 (s, 1H), 6.78 (s, 1H), 6.73 (s, 1H), 6.62 (d, J = 7.5 Hz, 1H), 3.67 (s, 3H), 3.66 (s, 3H), 2.91 - 2.86 (m, 2H), 2.66 - 2.61 (m, 1H), 2.44 - 2.37 (m, 4H), 2.35 (s, 6H), 2.24 - 2.14 (m, 2H), 1.81 - 1.75 (m, 2H); **^{13}C NMR** (100 MHz, $CDCl_3$): δ (ppm) 175.7, 173.3, 139.0, 137.7, 136.8, 136.4, 135.1, 133.9, 128.4, 127.9, 126.9, 126.2, 126.1, 125.9, 51.6, 51.5, 43.5, 37.0, 31.6, 28.4, 27.3, 26.6, 21.3.

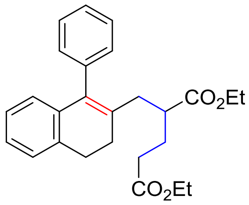

Diethyl 2-((1-phenyl-3,4-dihydronaphthalen-2-yl) methyl) pentanedioate (3ab).

Colorless oil, 97.5 mg, 0.240 mmol, 60% yield. ^{1}H-NMR (400 MHz, $CDCl_3$): δ (ppm) 7.44 - 7.40 (m, 2H), 7.37 - 7.33 (m 1H), 7.18 - 7.16 (m, 3H), 7.12 - 7.09 (m, 1H), 7.05 - 7.01 (m, 1H), 6.58 (d, *J* = 7.6 Hz, 1H), 4.17 - 4.08 (m, 4H), 2.90 (t, *J* = 8.3 Hz, 2H), 2.65 - 2.58 (m, 1H), 2.43 - 2.30 (m, 4H), 2.21 - 2.14 (m, 2H), 1.79 - 1.74 (m, 2H), 1.29 - 1.27 (m, 6H); ^{13}C-NMR (100 MHz, $CDCl_3$): δ (ppm) 175.1, 172.9, 139.2, 136.7, 136.2, 135.2, 134.5, 130.3, 128.4, 127.0, 126.8, 126.3, 126.2, 125.8, 60.4, 60.3, 43.5, 37.0, 31.8, 28.4, 27.4, 26.8, 14.2.

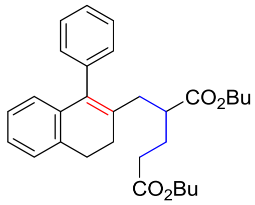

Dibutyl 2-((1-phenyl-3,4-dihydronaphthalen-2-yl) methyl) pentanedioate (3ac).

Colorless oil, 96.2 mg, 0.208 mmol, 52% yield.

^{1}H-NMR (400 MHz, $CDCl_3$): δ (ppm) 7.44 - 7.40 (m, 2H), 7.37 - 7.33 (m, 1H), 7.17 - 7.09 (m, 4H), 7.05 - 7.01 (m, 1H), 6.58 (d, *J* = 7.0 Hz, 1H), 4.07 - 4.03 (m, 4H), 2.91 (t, *J* = 8.1 Hz, 2H), 2.66 - 2.57 (m, 1H), 2.47 - 2.30 (m, 4H), 2.21 - 2.12 (m, 2H), 1.81 - 1.73 (m, 2H), 1.65 - 1.54 (m, 4H), 1.42 - 1.29 (m, 4H), 0.97 - 0.89 (m, 6H); ^{13}C-NMR (100 MHz, $CDCl_3$): δ (ppm) 175.1, 173.0, 139.2, 136.7, 136.2, 135.1, 134.4, 130.3, 128.4, 127.0, 126.8, 126.3, 126.2, 125.8, 64.4, 64.3, 43.5, 37.0, 31.9, 30.7, 30.6, 28.4, 27.4,26.8, 19.1 × 2, 13.7, 13.6.

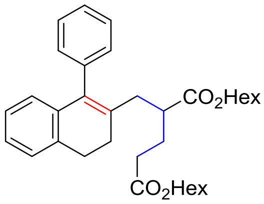

Dihexyl 2-((1-phenyl-3,4-dihydronaphthalen-2-yl) methyl) pentanedioate (3ad).

Colorless oil, 66.1 mg, 0.148 mmol, 37% yield.

^{1}H-NMR (400 MHz, $CDCl_3$): δ (ppm) 7.44 - 7.40 (m, 2H), 7.37 - 7.35 (m, 1H), 7.17 - 7.16 (m, 3H), 7.12 - 7.08 (m, 1H), 7.05 - 7.01 (m, 1H), 6.57 (d, *J* = 7.5 Hz, 1H), 4.09 - 4.02 (m, 4H), 2.92 - 2.88 (m, 2H), 2.64 - 2.62 (m, 1H), 2.45 - 2.32 (m, 4H), 2.22 - 2.16 (m, 2H), 1.79 - 1.71 (m, 2H), 1.65 - 1.56 (m, 6H), 1.32 - 1.28 (m, 10H), 0.93 - 0.87 (m, 6H); ^{13}C-NMR (100 MHz, $CDCl_3$): δ (ppm) 175.1, 173.0, 139.2, 136.7, 136.2, 135.1, 134.4, 130.3, 128.4, 127.0, 126.8, 126.3, 126.2, 125.8, 64.7, 64.6, 43.5, 37.0, 31.8, 31.4, 31.3, 28.6, 28.5, 28.4, 27.4, 26.7, 25.6 × 2, 22.5, 22.4, 14.0 ×2 .

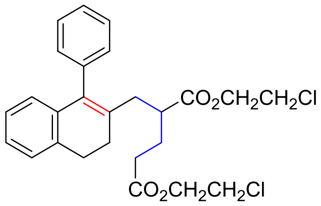

Bis(2-chloroethyl) 2-((1-phenyl-3,4-dihydronaphthalen -2-yl) methyl)pentanedioate (3ae).

Colorless oil, 89.2 mg, 0.200 mmol, 50.0% yield.

^{1}H-NMR (400 MHz, $CDCl_3$): δ (ppm) 7.45 - 7.43 (m, 2H), 7.38 - 7.36 (m, 1H), 7.18 - 7.10 (m, 4H), 7.06 - 7.02 (m, 1H), 6.58 (d, *J* = 7.6 Hz, 1H), 4.35 - 4.29 (m, 4H), 3.70 - 3.63 (m, 4H), 2.94 -2.89 (m, 2H), 2.73 - 2.67 (m, 1H), 2.50 - 2.33 (m, 4H), 2.31 - 2.26 (m, 2H), 1.84 - 1.79 (m, 2H); ^{13}C-NMR (100 MHz, $CDCl_3$) 174.6, 172.4, 139.1, 136.6, 136.4, 135.1, 134.0, 130.2, 128.4, 127.0, 126.9, 126.4, 126.2, 125.9, 64.0, 63.9, 43.2, 41.5, 41.4, 36.9, 31.5, 28.4, 27.4, 26.4.

3. NMR Spectra

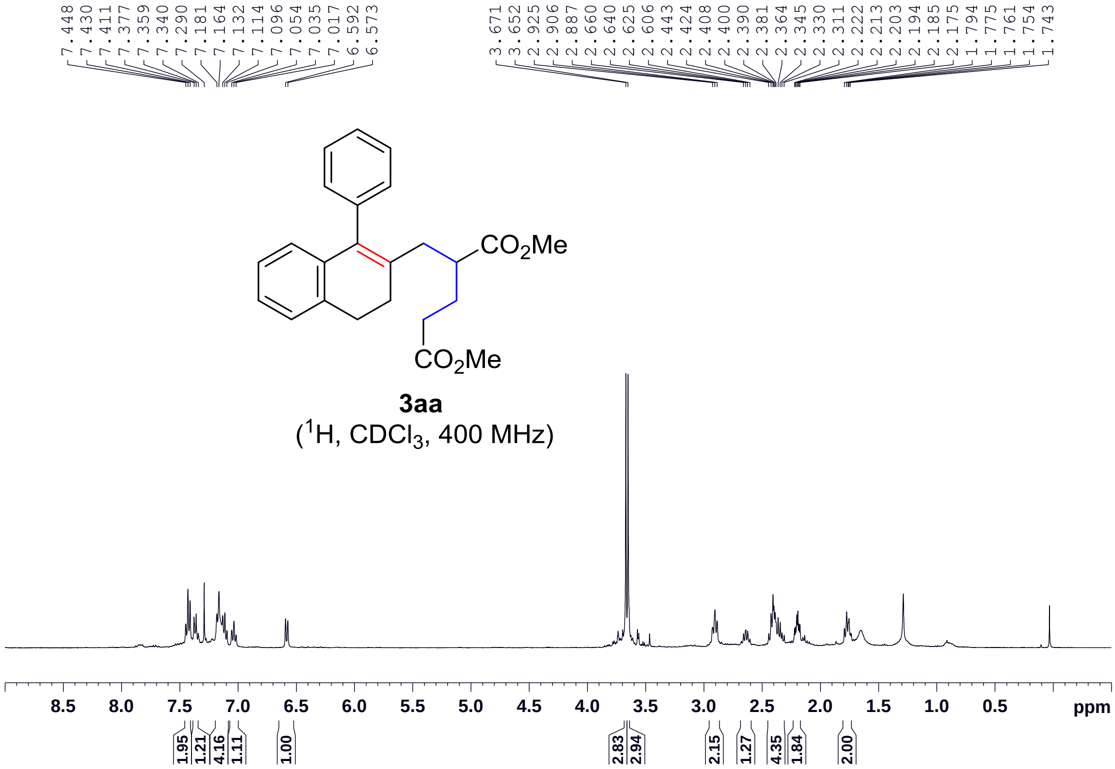

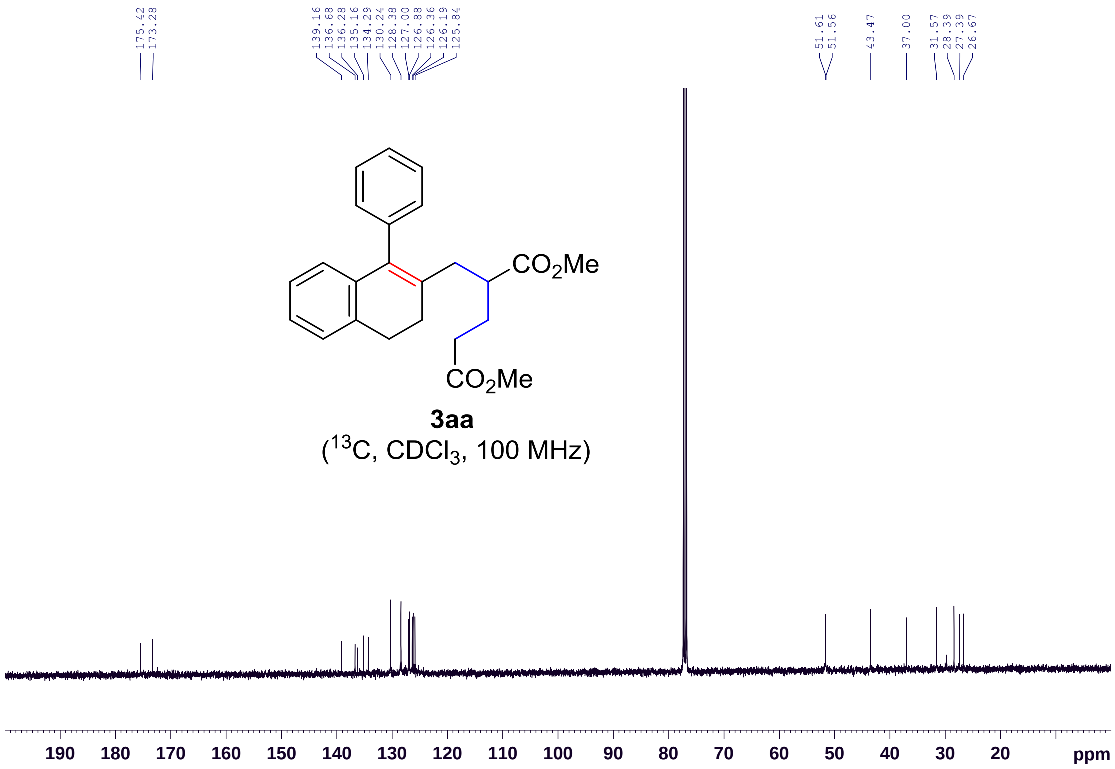

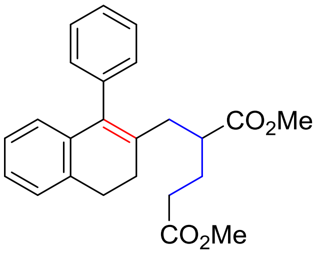
CO2Me
CO2Me

3aa

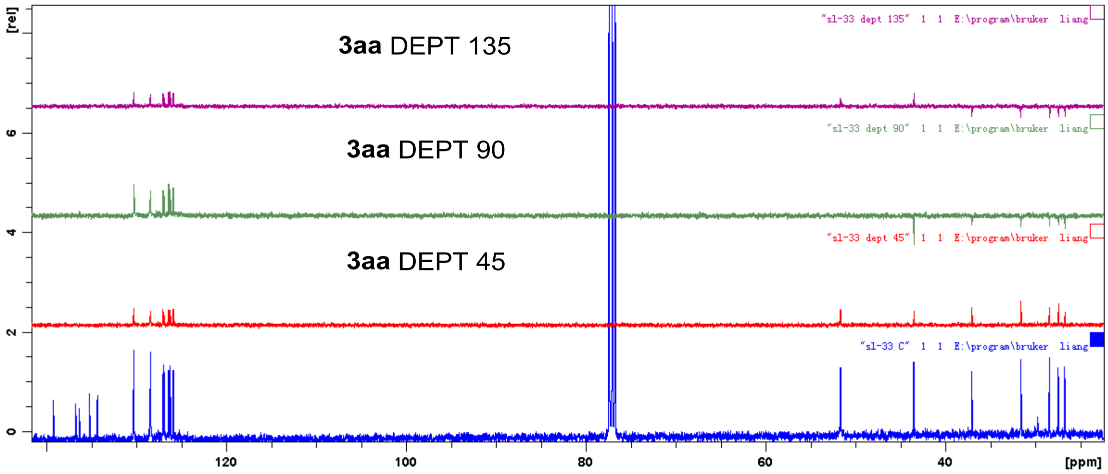
3aa DEPT 135
3aa DEPT 90
3aa DEPT 45
[rel]
[ppm]

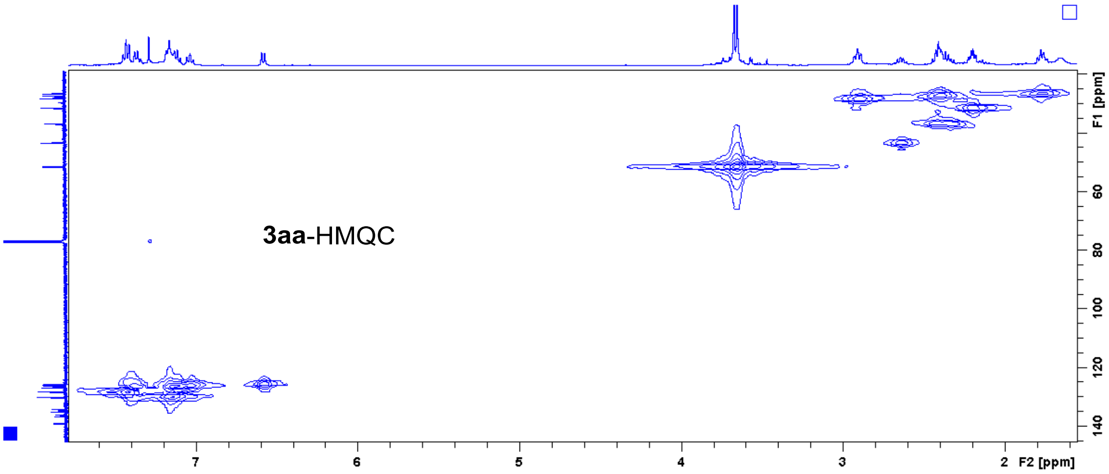
3aa-HMQC
F1 [ppm]
F2 [ppm]

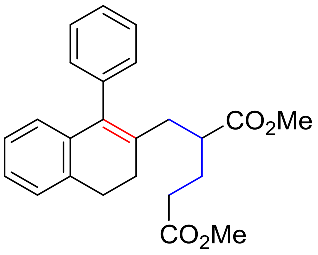
CO2Me
CO2Me

3aa

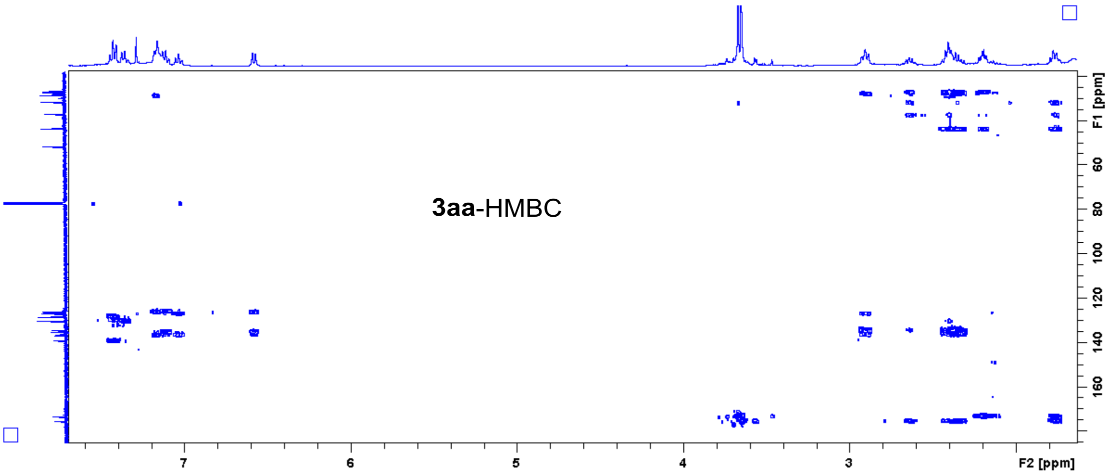
3aa-HMBC
F1 [ppm]
60
80
100
120
140
160
7
6
5
4
3
F2 [ppm]

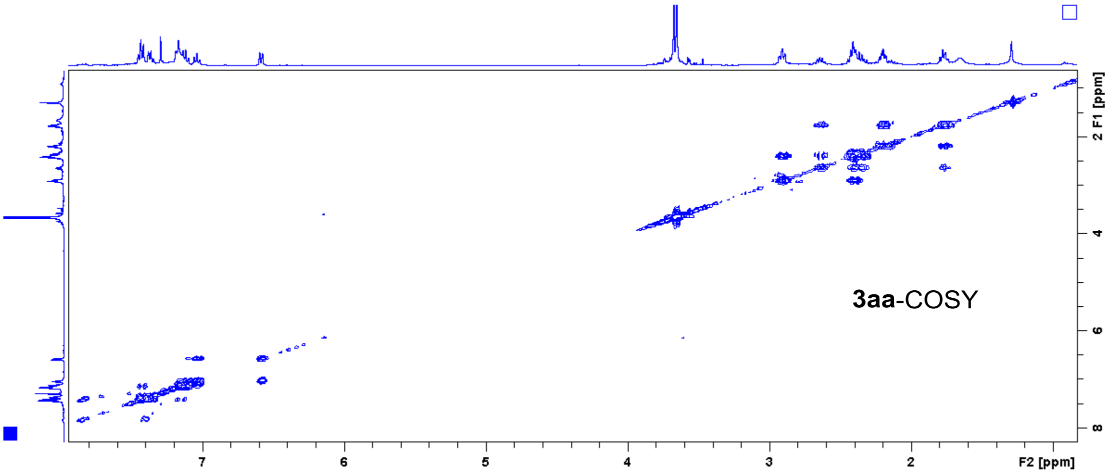
3aa-COSY
2 F1 [ppm]
4
6
8
7
6
5
4
3
2
F2 [ppm]

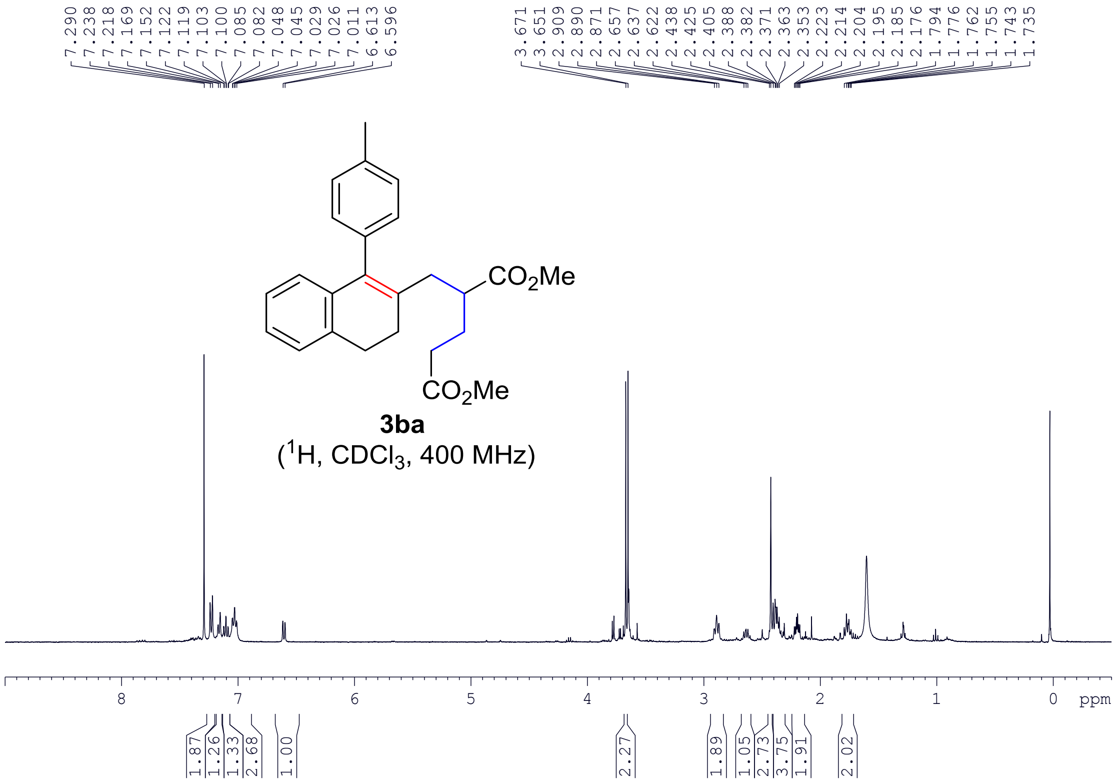
7.290
7.238
7.218
7.169
7.152
7.122
7.119
7.103
7.100
7.085
7.082
7.048
7.045
7.029
7.026
7.011
6.613
6.596
3.671
3.651
2.909
2.890
2.871
2.657
2.637
2.622
2.438
2.425
2.405
2.388
2.382
2.371
2.363
2.353
2.223
2.214
2.204
2.195
2.185
2.176
1.794
1.776
1.762
1.755
1.743
1.735
CO2Me
CO2Me
3ba
(1H, CDCl3, 400 MHz)
8
7
6
5
4
3
2
1
0
ppm
1.87
1.26
1.33
2.68
1.00
2.27
1.89
1.05
2.73
3.75
1.91
2.02

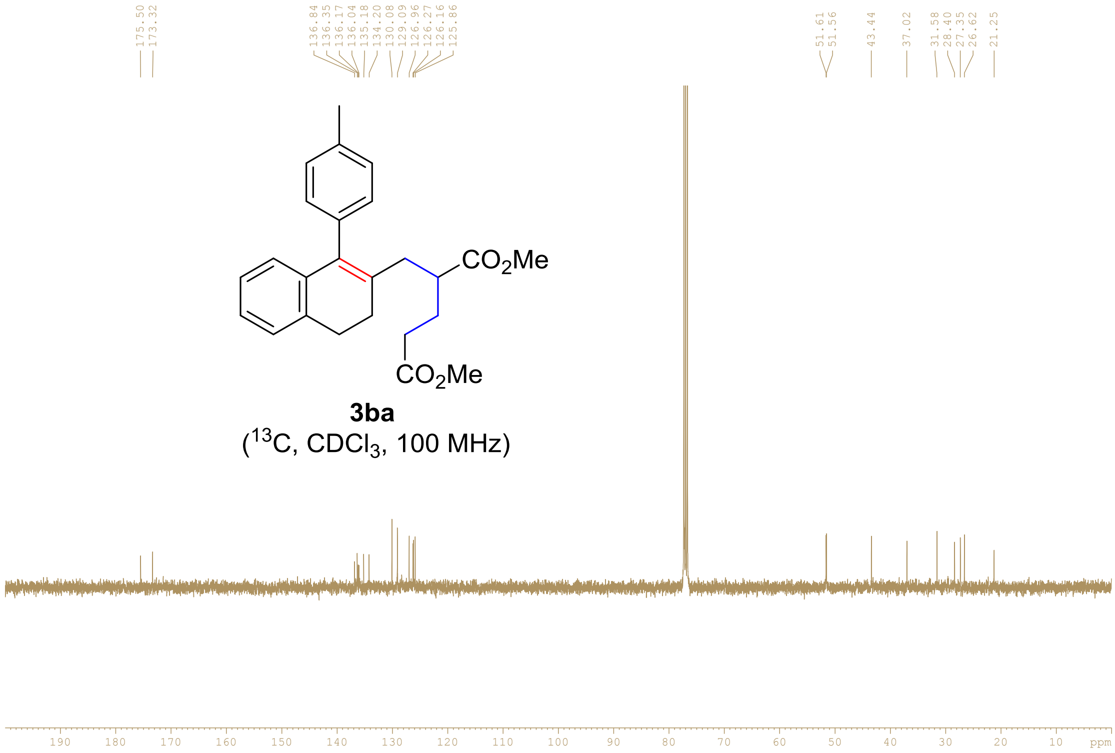
175.50
173.32
136.84
136.35
136.17
136.04
135.18
134.20
130.08
129.09
126.96
126.27
126.16
125.86
51.61
51.56
43.44
37.02
31.58
28.40
27.35
26.62
21.25
CO2Me
CO2Me
3ba
(13C, CDCl3, 100 MHz)
190
180
170
160
150
140
130
120
110
100
90
80
70
60
50
40
30
20
10
ppm

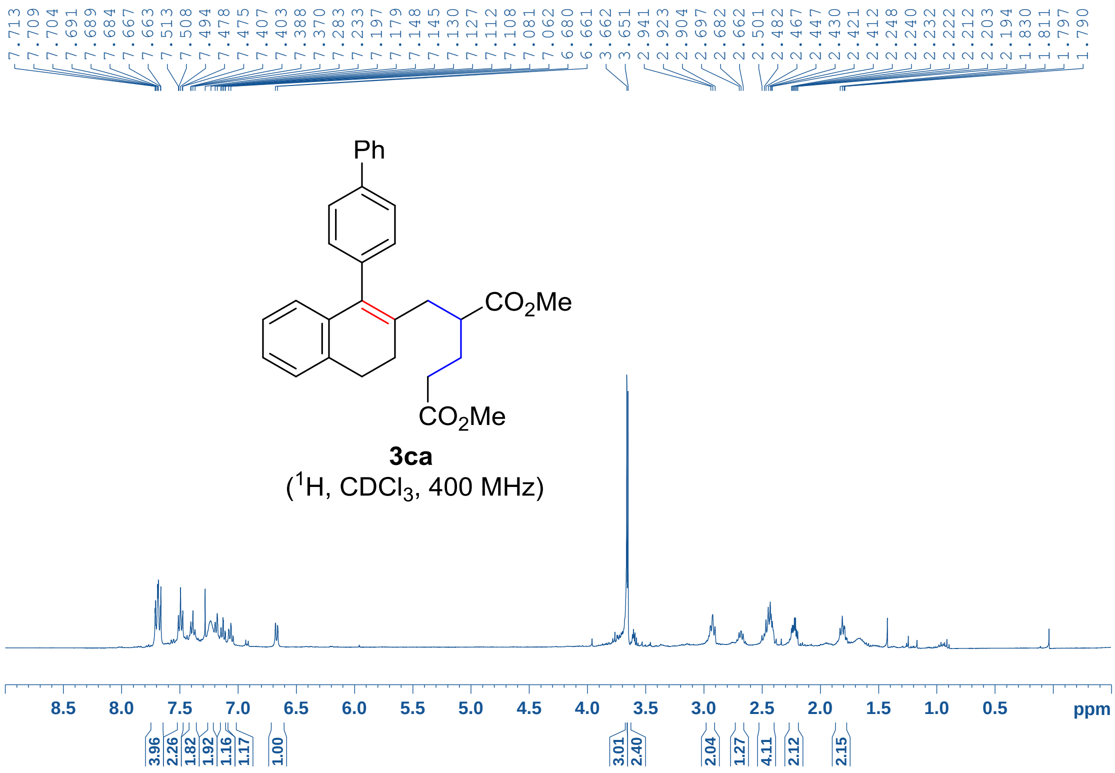
7.713 7.709 7.704 7.691 7.689 7.684 7.667 7.663 7.513 7.508 7.494 7.478 7.475 7.407 7.403 7.388 7.370 7.283 7.233 7.197 7.179 7.148 7.145 7.130 7.127 7.112 7.108 7.081 7.062 6.680 6.661 3.662 3.651 2.941 2.923 2.904 2.697 2.682 2.662 2.501 2.482 2.467 2.447 2.430 2.421 2.412 2.248 2.240 2.232 2.222 2.212 2.203 2.194 1.830 1.811 1.797 1.790
Ph
CO2Me
CO2Me
3ca
(1H, CDCl3, 400 MHz)
8.5 8.0 7.5 7.0 6.5 6.0 5.5 5.0 4.5 4.0 3.5 3.0 2.5 2.0 1.5 1.0 0.5 ppm
3.96 2.26 1.82 1.92 1.16 1.17 1.00 3.01 2.40 2.04 1.27 4.11 2.12 2.15

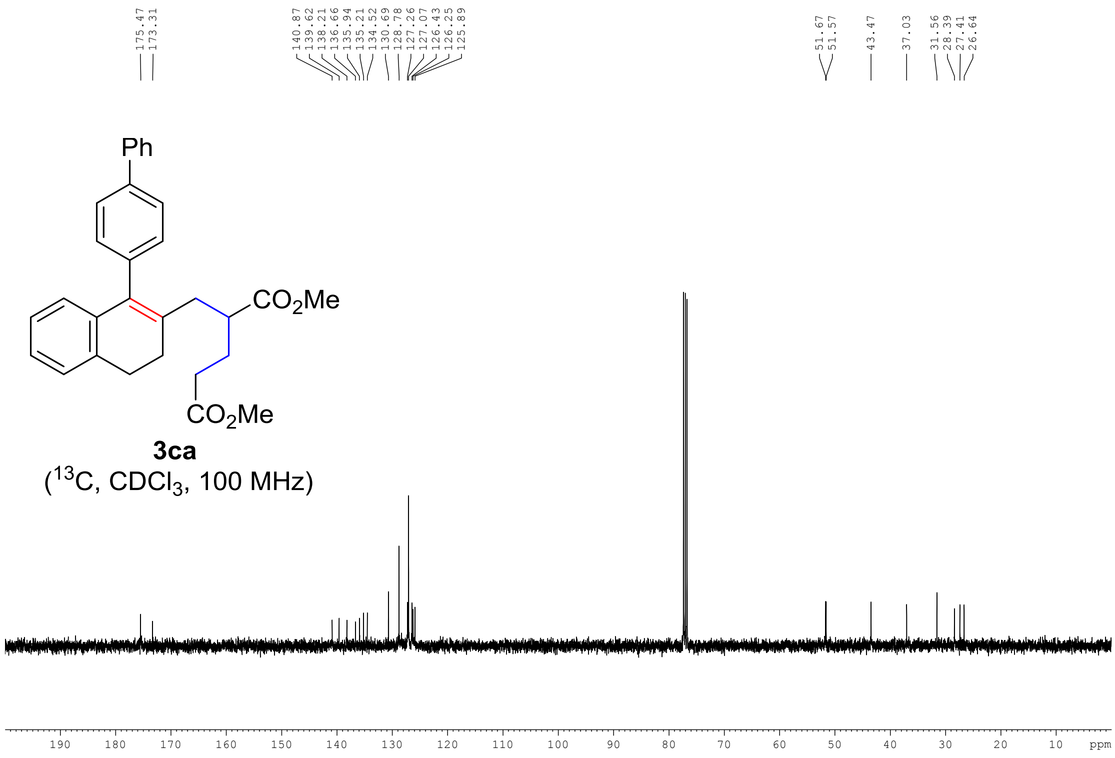
175.47 173.31 140.87 139.62 138.21 136.66 135.94 135.21 134.52 130.69 128.78 127.26 127.07 126.43 126.25 125.89 51.67 51.57 43.47 37.03 31.56 28.39 27.41 26.64
Ph
CO2Me
CO2Me
3ca
(13C, CDCl3, 100 MHz)
190 180 170 160 150 140 130 120 110 100 90 80 70 60 50 40 30 20 10 ppm

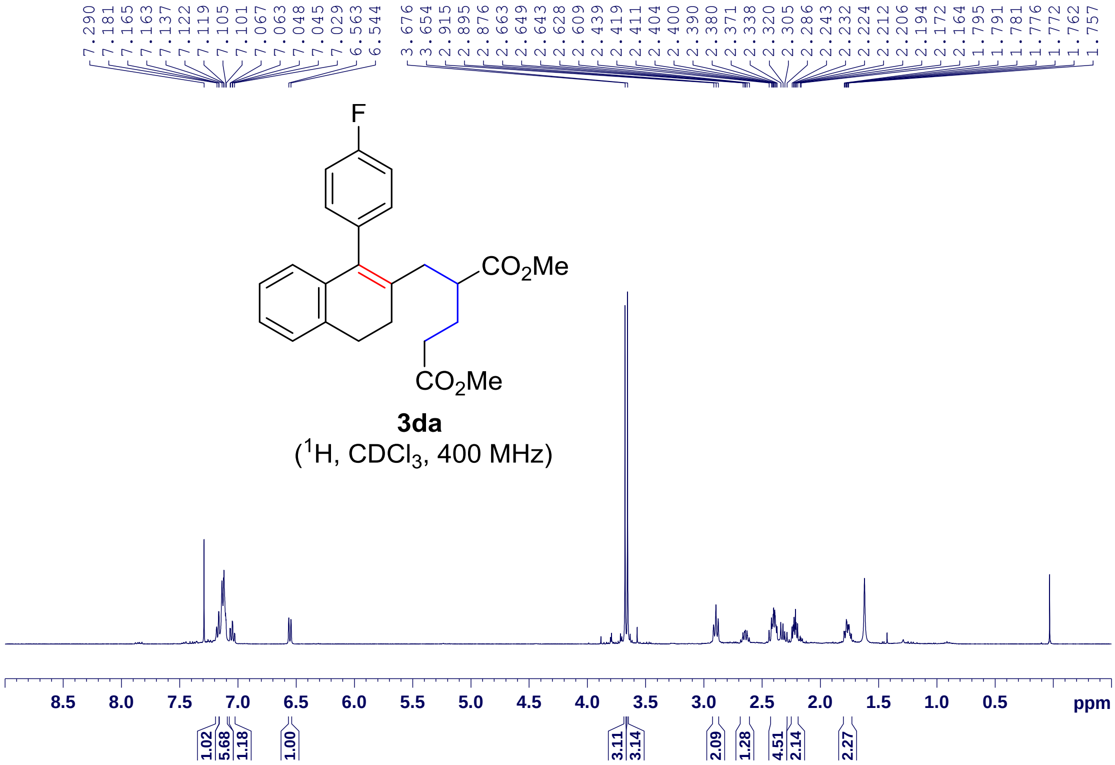
7.290 7.181 7.165 7.163 7.137 7.122 7.119 7.105 7.101 7.067 7.063 7.048 7.045 7.029 6.563 6.544
3.676 3.654 2.915 2.895 2.876 2.663 2.649 2.643 2.628 2.609 2.439 2.419 2.411 2.404 2.400 2.390 2.380 2.371 2.338 2.320 2.305 2.286 2.243 2.232 2.224 2.212 2.206 2.194 2.172 2.164 1.795 1.791 1.781 1.776 1.772 1.762 1.757
F
CO_2Me
CO_2Me
3da
(^{1}H, $CDCl_3$, 400 MHz)
8.5 8.0 7.5 7.0 6.5 6.0 5.5 5.0 4.5 4.0 3.5 3.0 2.5 2.0 1.5 1.0 0.5 ppm
1.02 5.68 1.18 1.00 3.11 3.14 2.09 1.28 4.51 2.14 2.27

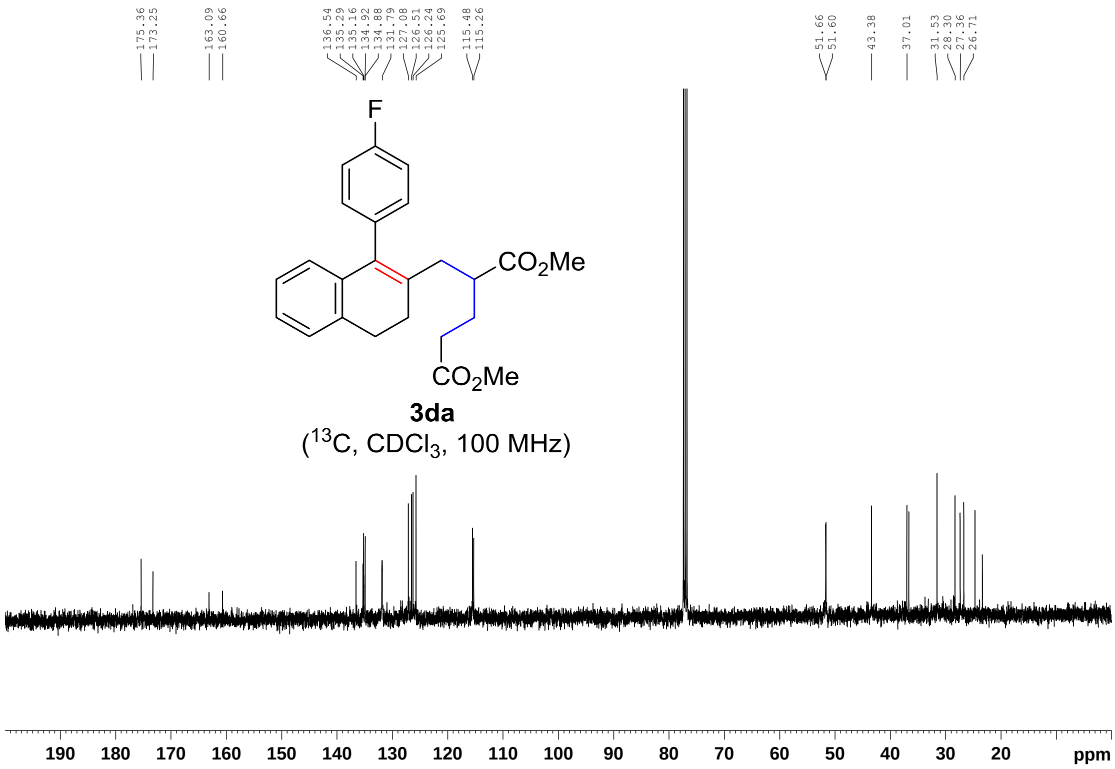
175.36 173.25 163.09 160.66 136.54 135.29 135.16 134.92 134.88 131.79 127.08 126.51 126.24 125.69 115.48 115.26 51.66 51.60 43.38 37.01 31.53 28.30 27.36 26.71
F
CO_2Me
CO_2Me
3da
(^{13}C, $CDCl_3$, 100 MHz)
190 180 170 160 150 140 130 120 110 100 90 80 70 60 50 40 30 20 ppm

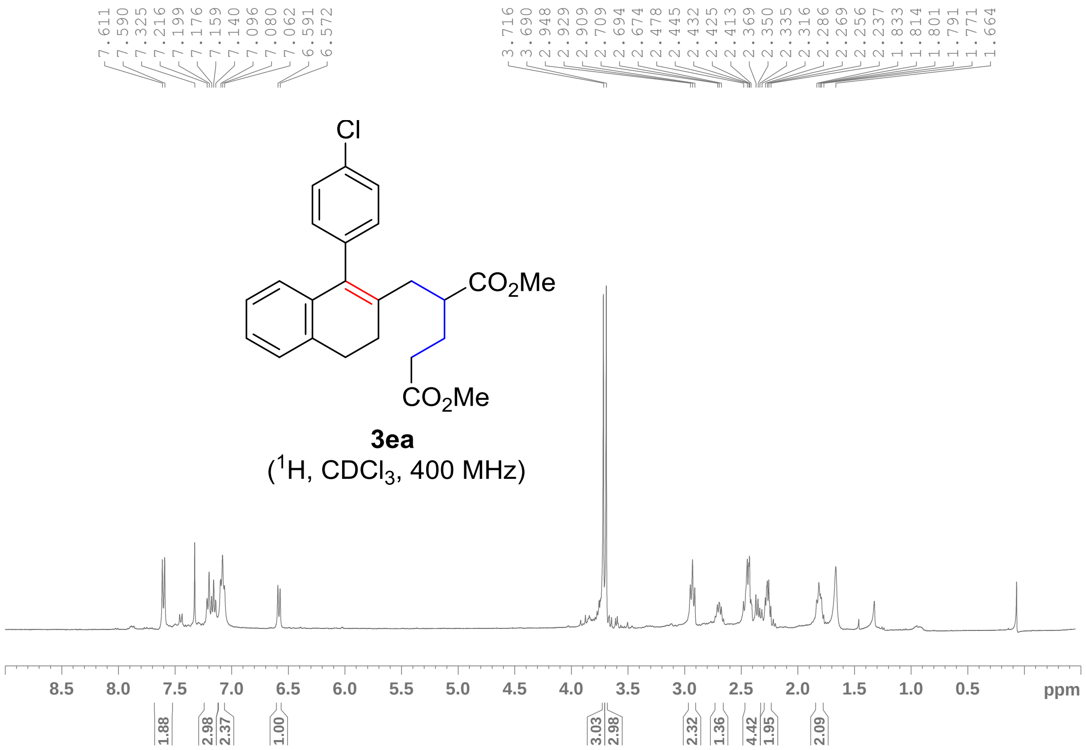

3ea

(^{1}H, $CDCl_3$, 400 MHz)

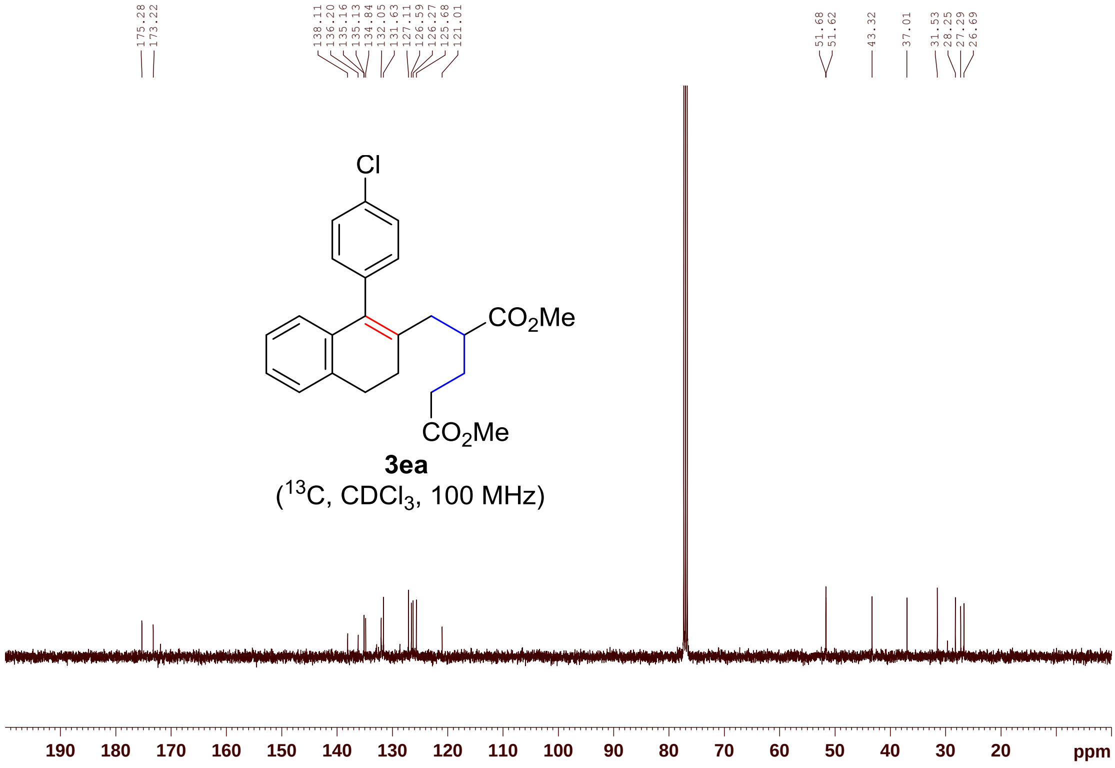

3ea

(^{13}C, $CDCl_3$, 100 MHz)

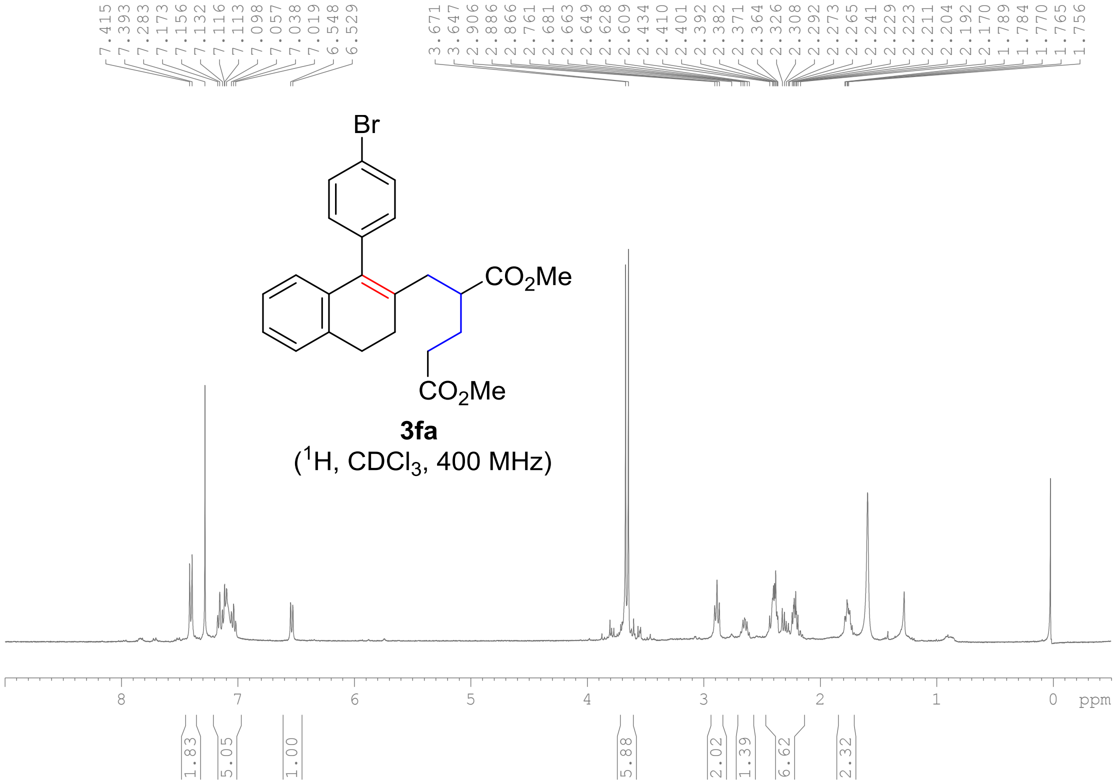
7.415 7.393 7.283 7.173 7.156 7.132 7.116 7.113 7.098 7.057 7.038 7.019 6.548 6.529
3.671 3.647 2.906 2.886 2.866 2.761 2.681 2.663 2.649 2.628 2.609 2.434 2.410 2.401 2.392 2.382 2.371 2.364 2.326 2.308 2.292 2.273 2.265 2.241 2.229 2.223 2.211 2.204 2.192 2.170 1.789 1.784 1.770 1.765 1.756
Br
CO_2Me
CO_2Me
3fa
(^{1}H, $CDCl_3$, 400 MHz)
8 7 6 5 4 3 2 1 0 ppm
1.83 5.05 1.00 5.88 2.02 1.39 6.62 2.32

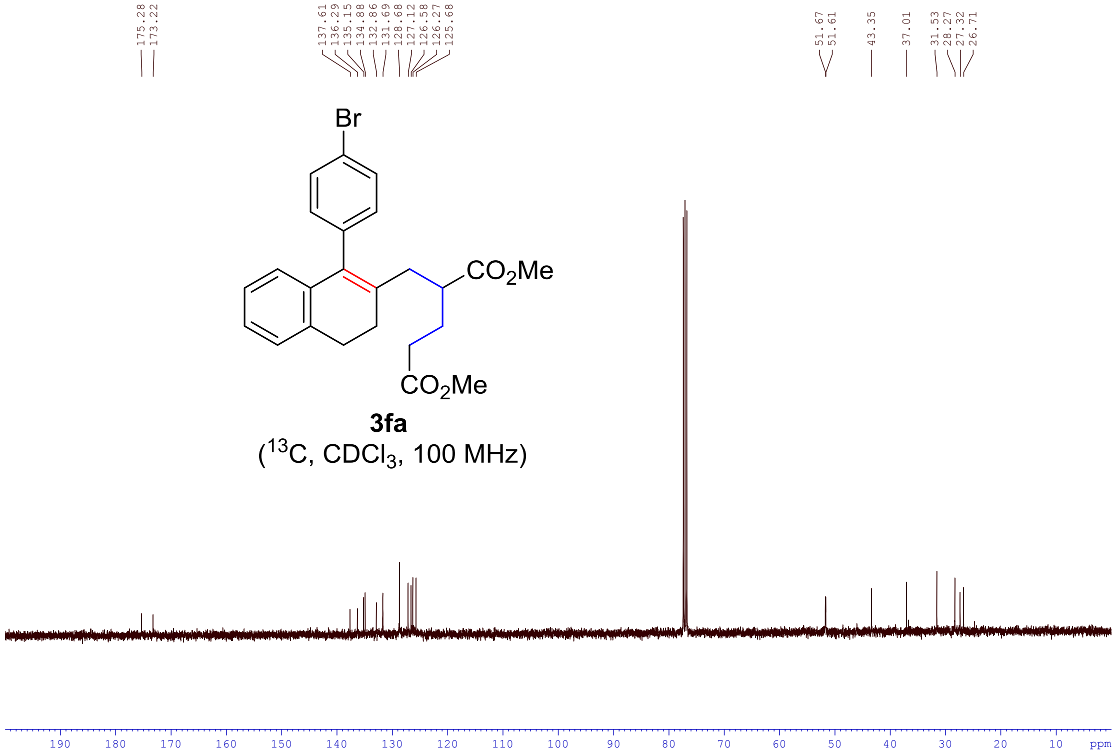
175.28 173.22
137.61 136.29 135.15 134.88 132.86 131.69 128.68 127.12 126.58 126.27 125.68
51.67 51.61 43.35 37.01 31.53 28.27 27.32 26.71
Br
CO_2Me
CO_2Me
3fa
(^{13}C, $CDCl_3$, 100 MHz)
190 180 170 160 150 140 130 120 110 100 90 80 70 60 50 40 30 20 10 ppm

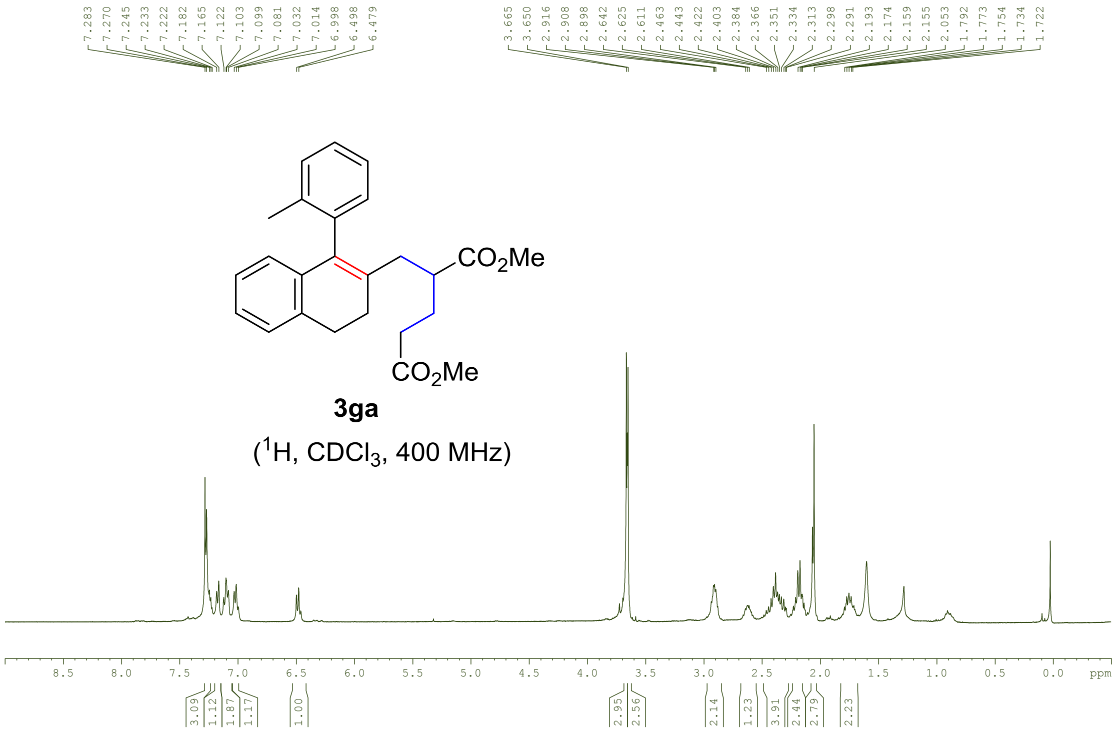
7.283 7.270 7.245 7.233 7.222 7.182 7.165 7.122 7.103 7.099 7.081 7.032 7.014 6.998 6.498 6.479
3.665 3.650 2.916 2.908 2.898 2.642 2.625 2.611 2.463 2.443 2.422 2.403 2.384 2.366 2.351 2.334 2.313 2.298 2.291 2.193 2.174 2.159 2.155 2.053 1.792 1.773 1.754 1.734 1.722
CO2Me
CO2Me
3ga
(1H, CDCl3, 400 MHz)
3.09 1.12 1.87 1.17 1.00 2.95 2.56 2.14 1.23 3.91 2.44 2.79 2.23
ppm

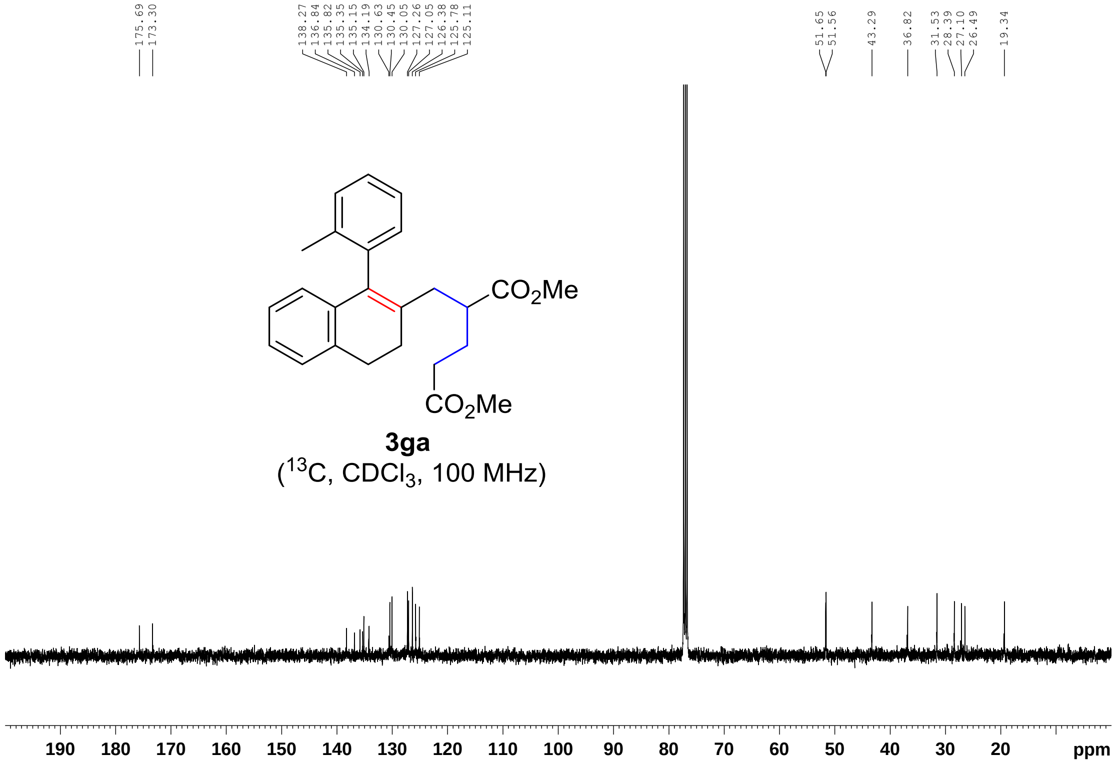
175.69 173.30
138.27 136.84 135.82 135.35 135.15 134.19 130.63 130.45 130.05 127.26 127.05 126.38 125.78 125.11
51.65 51.56 43.29 36.82 31.53 28.39 27.10 26.49 19.34
CO2Me
CO2Me
3ga
(13C, CDCl3, 100 MHz)
190 180 170 160 150 140 130 120 110 100 90 80 70 60 50 40 30 20 ppm

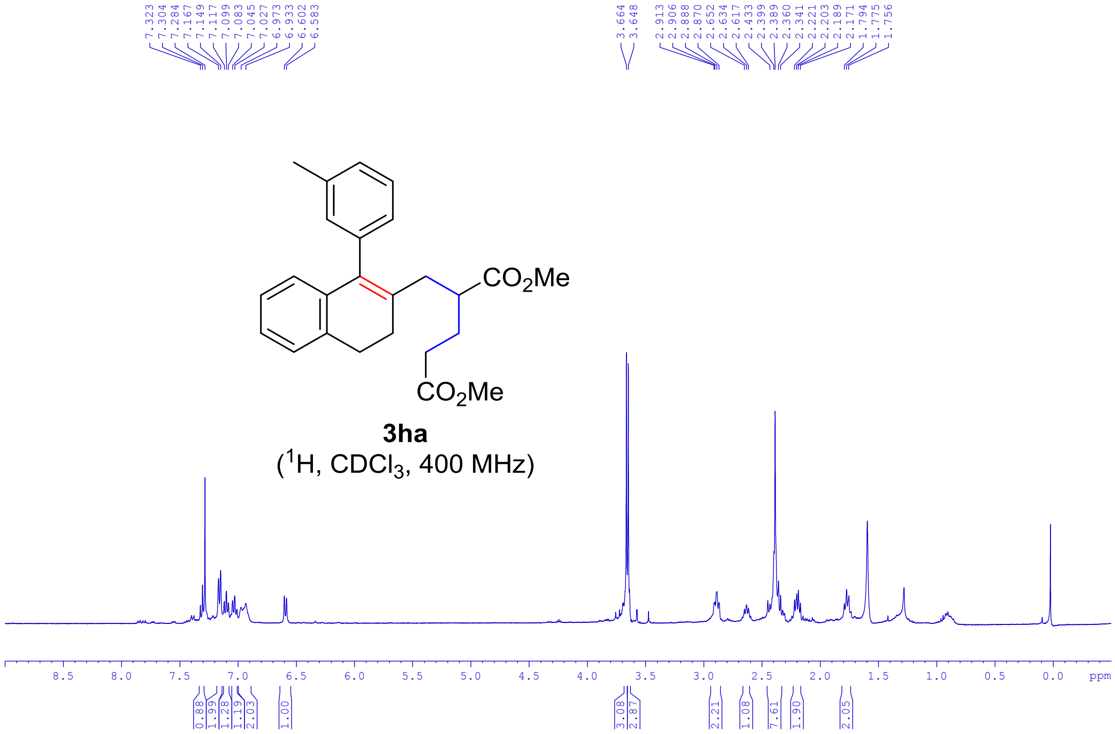

7.323 7.304 7.284 7.167 7.149 7.117 7.099 7.083 7.045 7.027 6.973 6.933 6.602 6.583
3.664 3.648
2.913 2.906 2.888 2.870 2.652 2.634 2.617 2.433 2.399 2.389 2.360 2.341 2.221 2.203 2.189 2.171 1.794 1.775 1.756
CO2Me
CO2Me
3ha
(1H, CDCl3, 400 MHz)
8.5 8.0 7.5 7.0 6.5 6.0 5.5 5.0 4.5 4.0 3.5 3.0 2.5 2.0 1.5 1.0 0.5 0.0 ppm
0.88 1.91 1.28 1.19 2.03 1.00 3.08 2.87 2.21 1.08 7.61 1.90 2.05

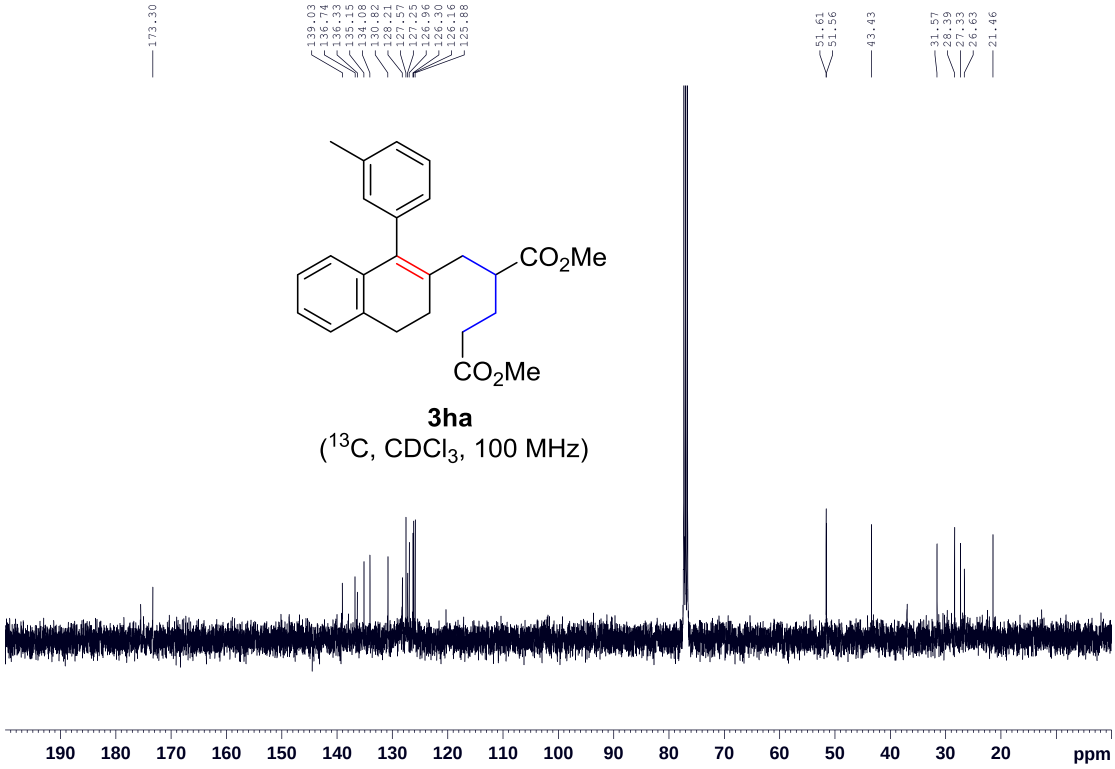

173.30
139.03 136.74 136.33 135.15 134.08 130.82 128.21 127.57 127.25 126.96 126.30 126.16 125.88
51.61 51.56
43.43
31.57 28.39 27.33 26.63 21.46
CO2Me
CO2Me
3ha
(13C, CDCl3, 100 MHz)
190 180 170 160 150 140 130 120 110 100 90 80 70 60 50 40 30 20 ppm

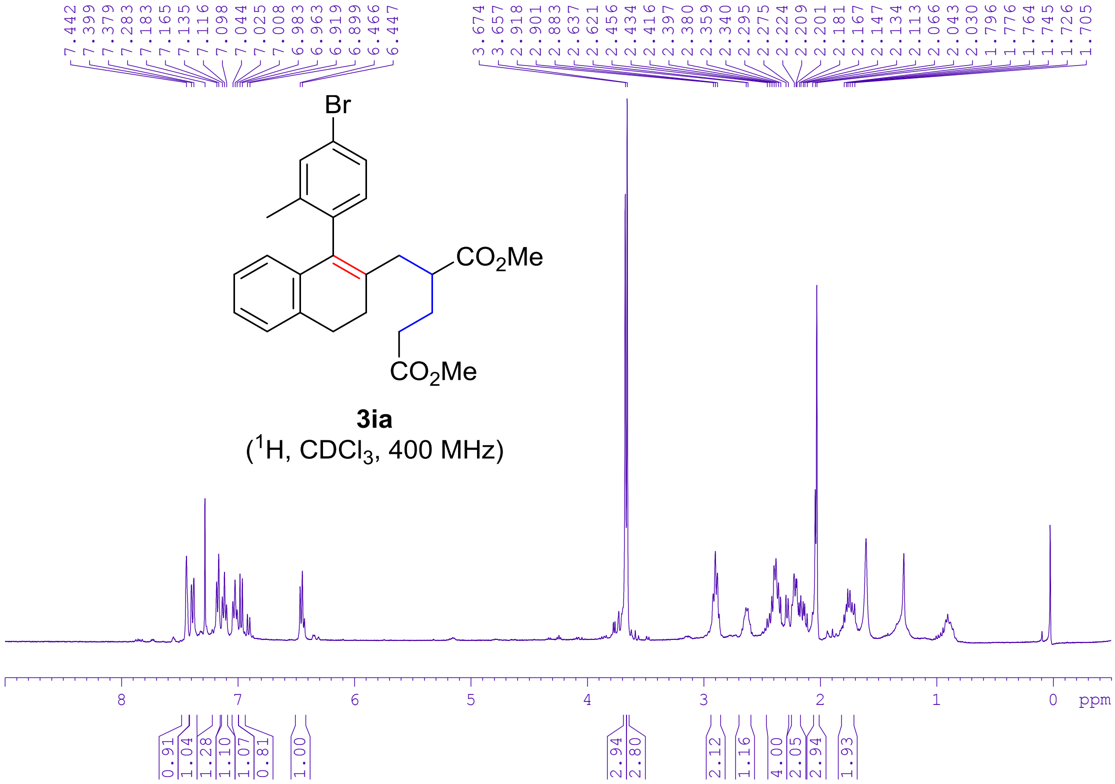
Br
CO2Me
CO2Me
3ia
(1H, CDCl3, 400 MHz)
ppm

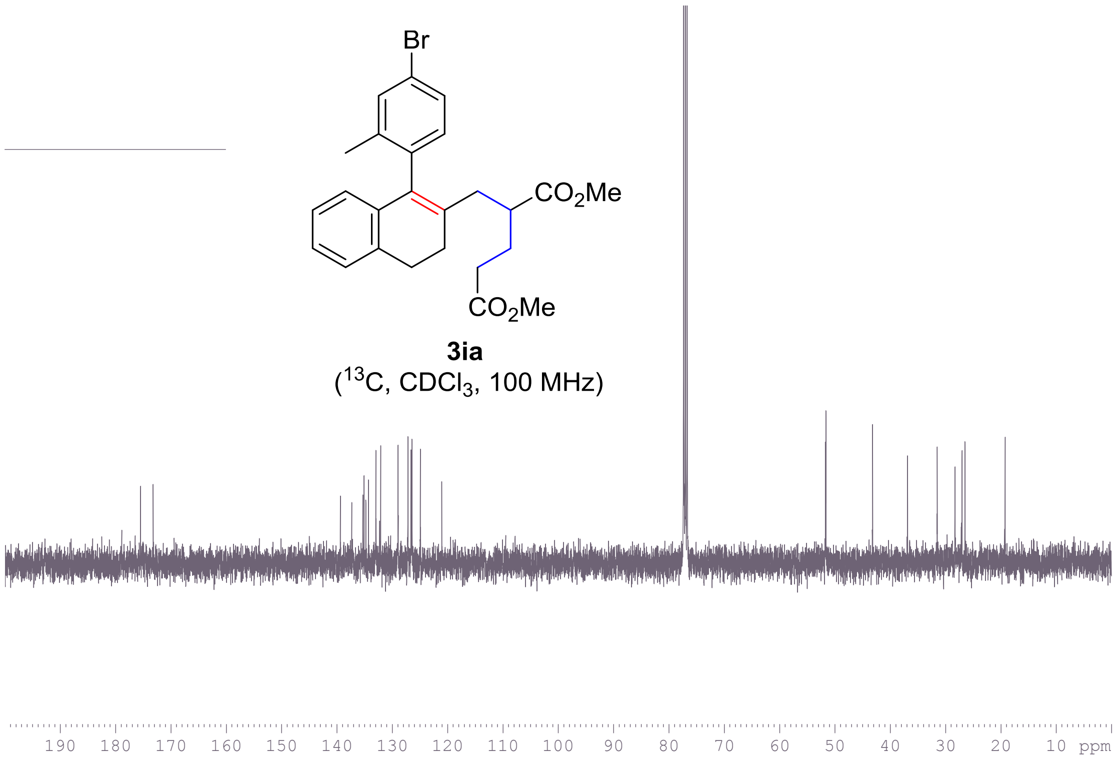
Br
CO2Me
CO2Me
3ia
(13C, CDCl3, 100 MHz)
ppm

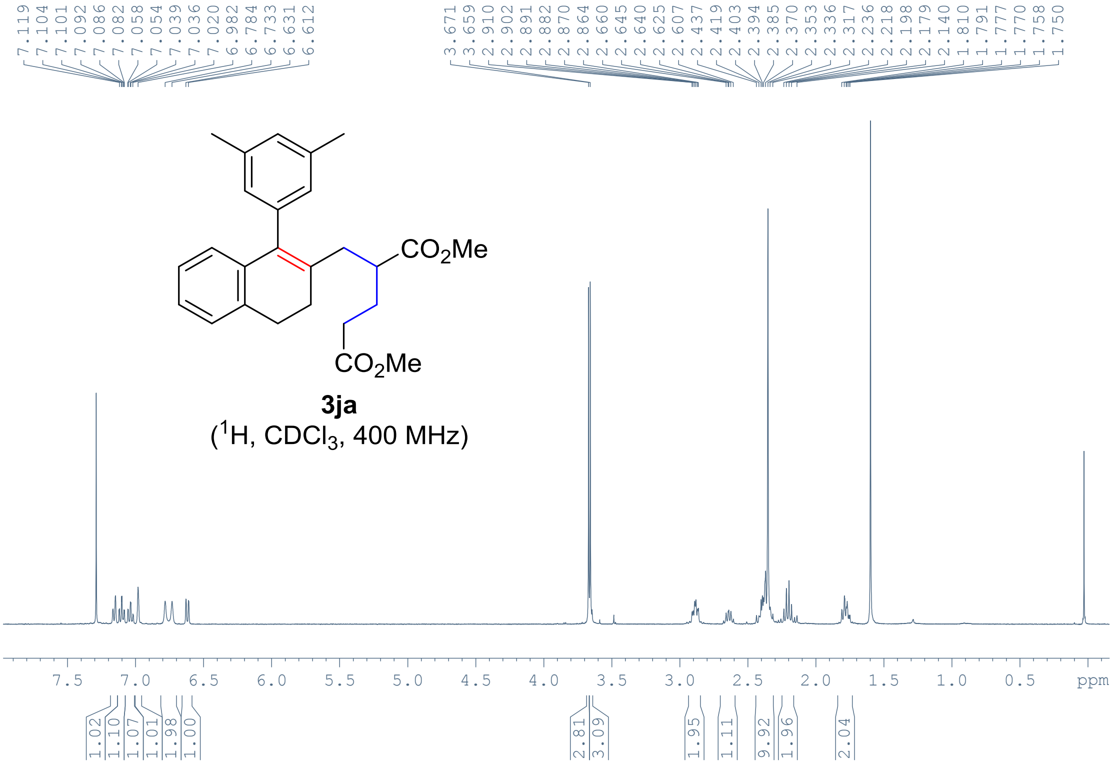
7.119 7.104 7.101 7.092 7.086 7.082 7.058 7.054 7.039 7.036 7.020 6.982 6.784 6.733 6.631 6.612
3.671 3.659 2.910 2.902 2.891 2.882 2.870 2.864 2.660 2.645 2.640 2.625 2.607 2.437 2.419 2.403 2.394 2.385 2.370 2.353 2.336 2.317 2.236 2.218 2.198 2.179 2.140 1.810 1.791 1.777 1.770 1.758 1.750
CO_2Me
CO_2Me
3ja
(^1H, $CDCl_3$, 400 MHz)
7.5 7.0 6.5 6.0 5.5 5.0 4.5 4.0 3.5 3.0 2.5 2.0 1.5 1.0 0.5 ppm
1.02 1.10 1.07 1.01 1.98 1.00 2.81 3.09 1.95 1.11 9.92 1.96 2.04

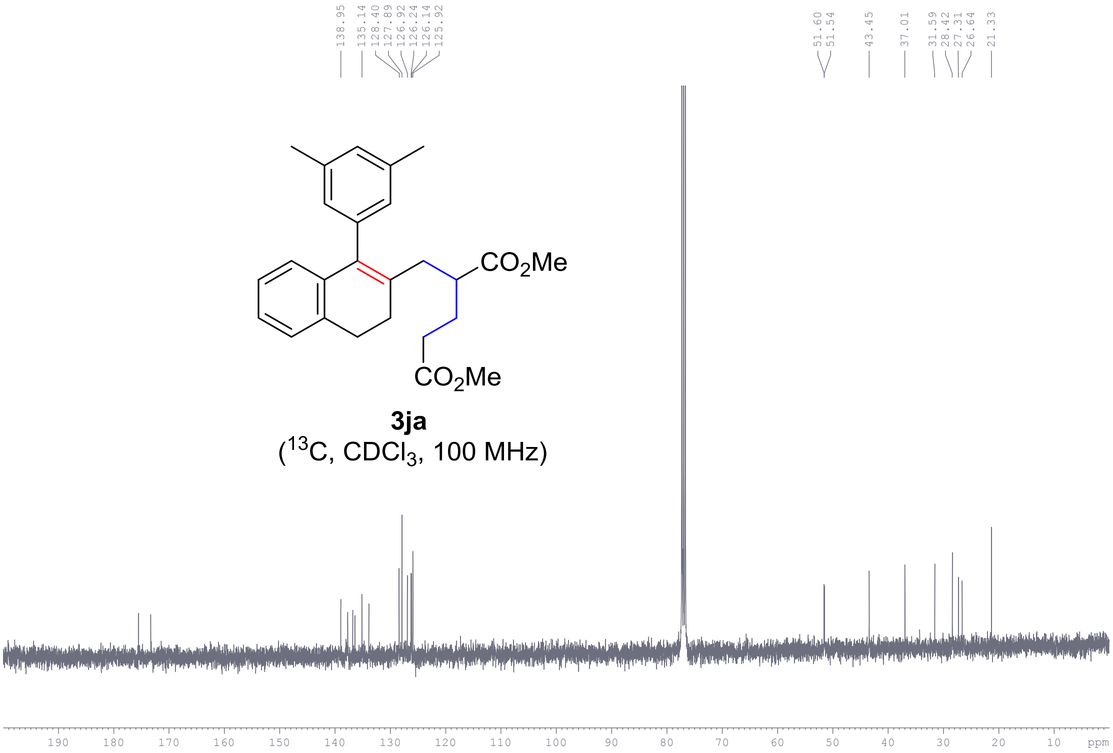
138.95 135.14 128.40 127.89 126.92 126.24 126.14 125.92
51.60 51.54 43.45 37.01 31.59 28.42 27.31 26.64 21.33
CO_2Me
CO_2Me
3ja
(^{13}C, $CDCl_3$, 100 MHz)
190 180 170 160 150 140 130 120 110 100 90 80 70 60 50 40 30 20 10 ppm

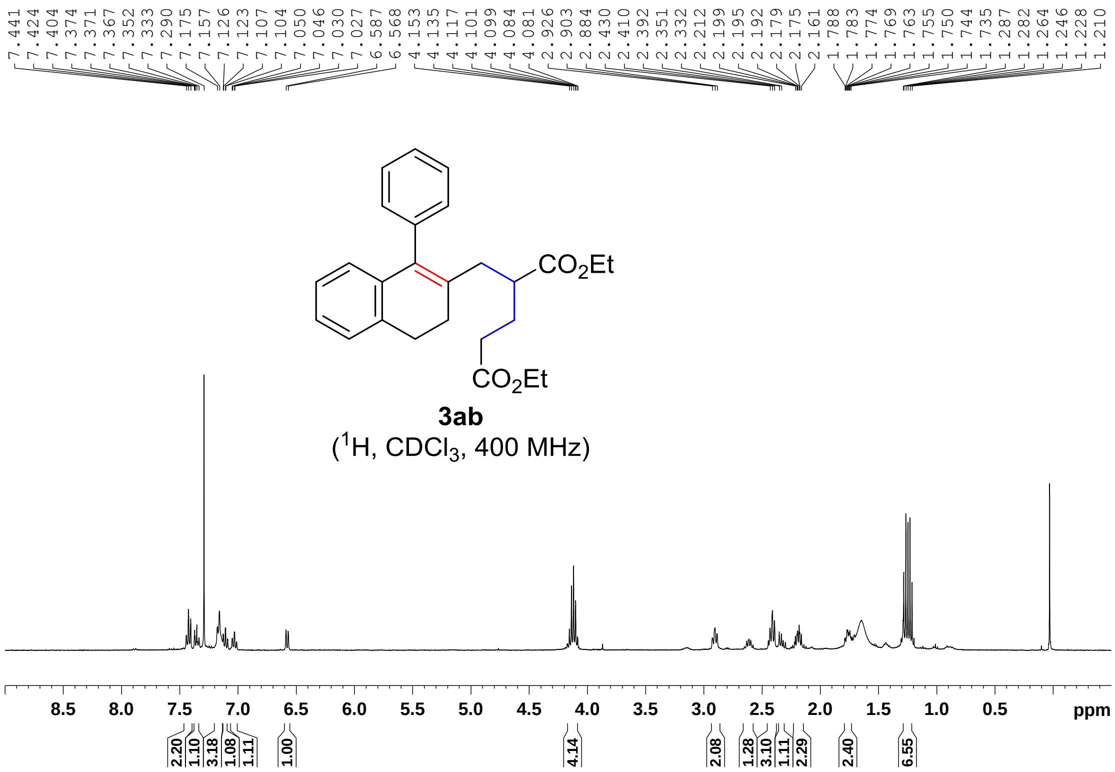
7.441 7.424 7.404 7.374 7.371 7.367 7.352 7.333 7.290 7.175 7.157 7.126 7.123 7.107 7.104 7.050 7.046 7.030 7.027 6.587 6.568 4.153 4.135 4.117 4.101 4.099 4.084 4.081 2.926 2.903 2.884 2.430 2.410 2.392 2.351 2.332 2.212 2.199 2.195 2.192 2.179 2.175 2.161 1.788 1.783 1.774 1.769 1.763 1.755 1.750 1.744 1.735 1.287 1.282 1.264 1.246 1.228 1.210
CO2Et
CO2Et
3ab
(1H, CDCl3, 400 MHz)
8.5 8.0 7.5 7.0 6.5 6.0 5.5 5.0 4.5 4.0 3.5 3.0 2.5 2.0 1.5 1.0 0.5 ppm
2.20 1.10 3.18 1.08 1.11 1.00 4.14 2.08 1.28 3.10 1.11 2.29 2.40 6.55

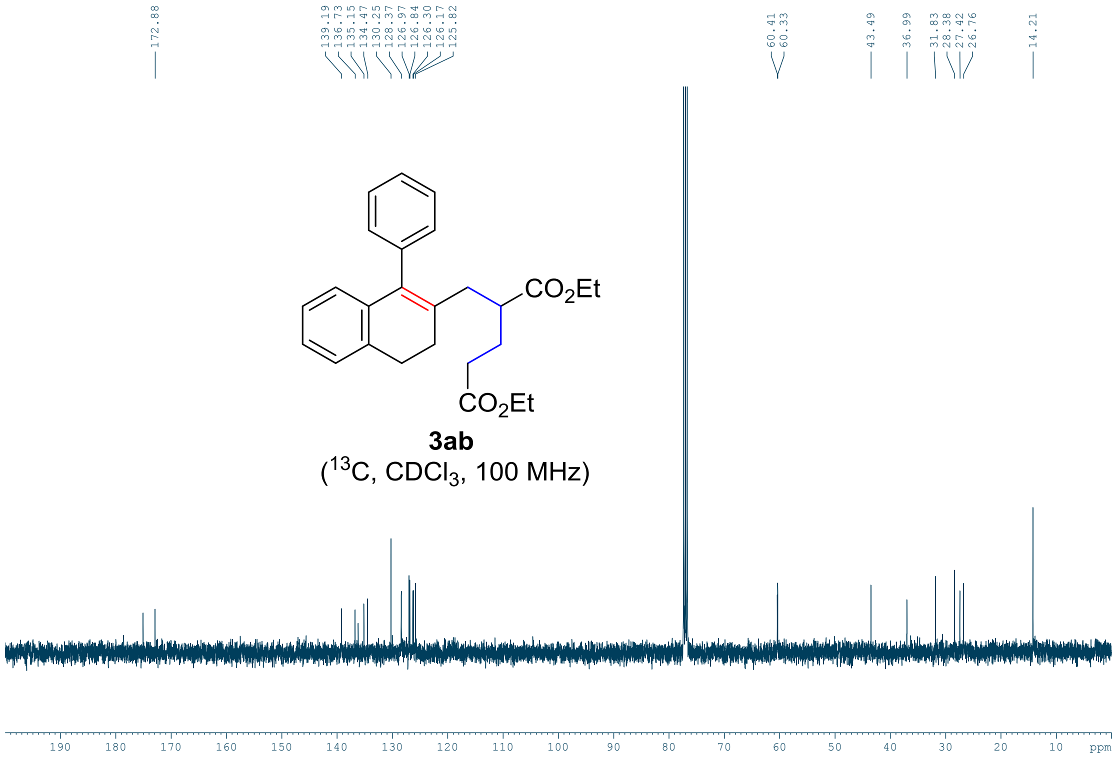
172.88
139.19 136.73 135.15 134.47 130.25 128.37 126.97 126.84 126.30 126.17 125.82
60.41 60.33
43.49
36.99
31.83 28.38 27.42 26.76
14.21
CO2Et
CO2Et
3ab
(13C, CDCl3, 100 MHz)
190 180 170 160 150 140 130 120 110 100 90 80 70 60 50 40 30 20 10 ppm

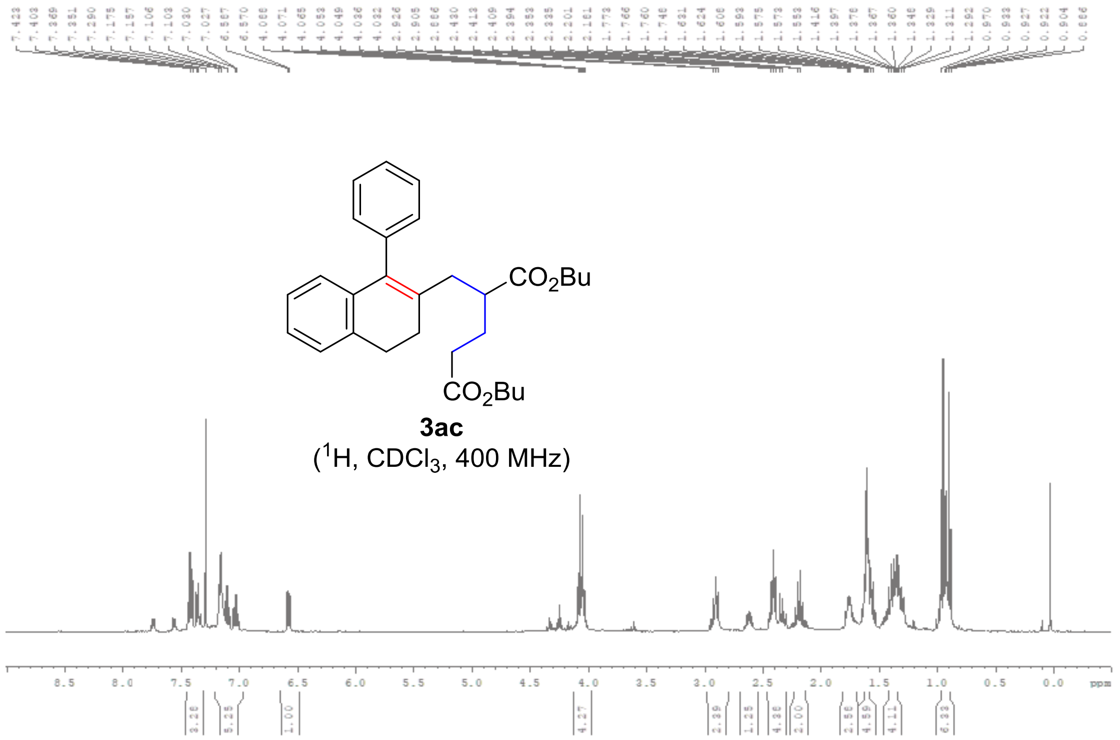
CO2Bu
CO2Bu
3ac
(1H, CDCl3, 400 MHz)

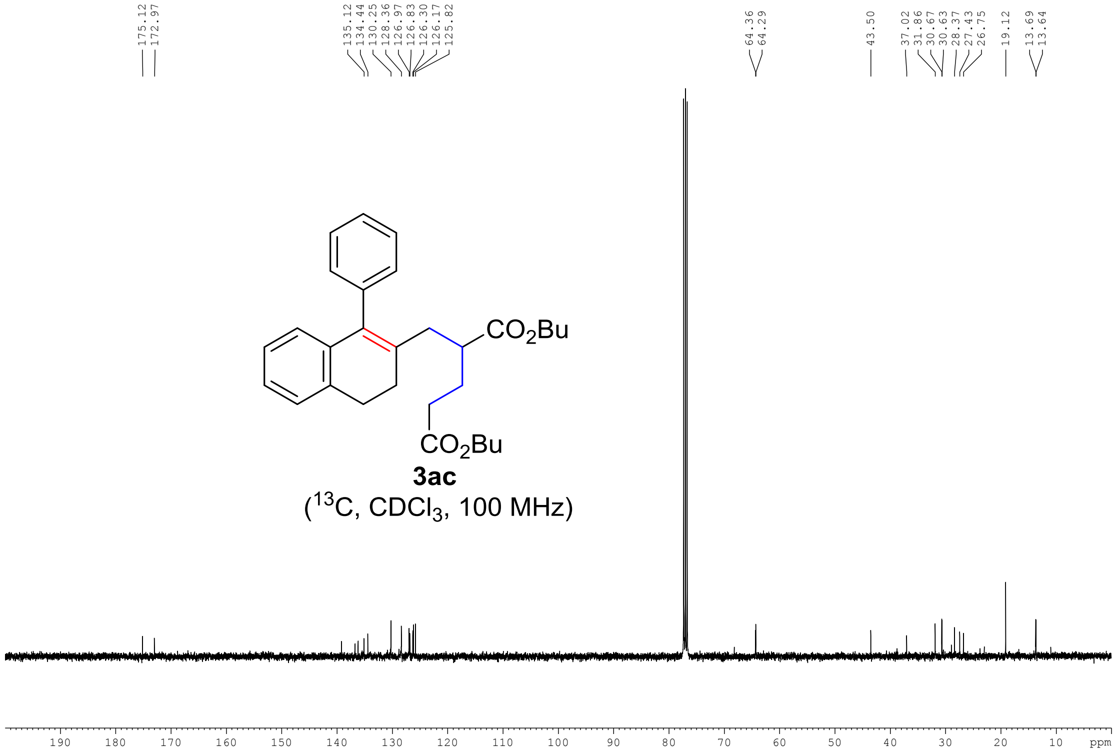
175.12
172.97
135.12
134.44
130.25
128.36
126.97
126.83
126.30
126.17
125.82
64.36
64.29
43.50
37.02
31.86
30.67
30.63
28.37
27.43
26.75
19.12
13.69
13.64
CO2Bu
CO2Bu
3ac
(13C, CDCl3, 100 MHz)

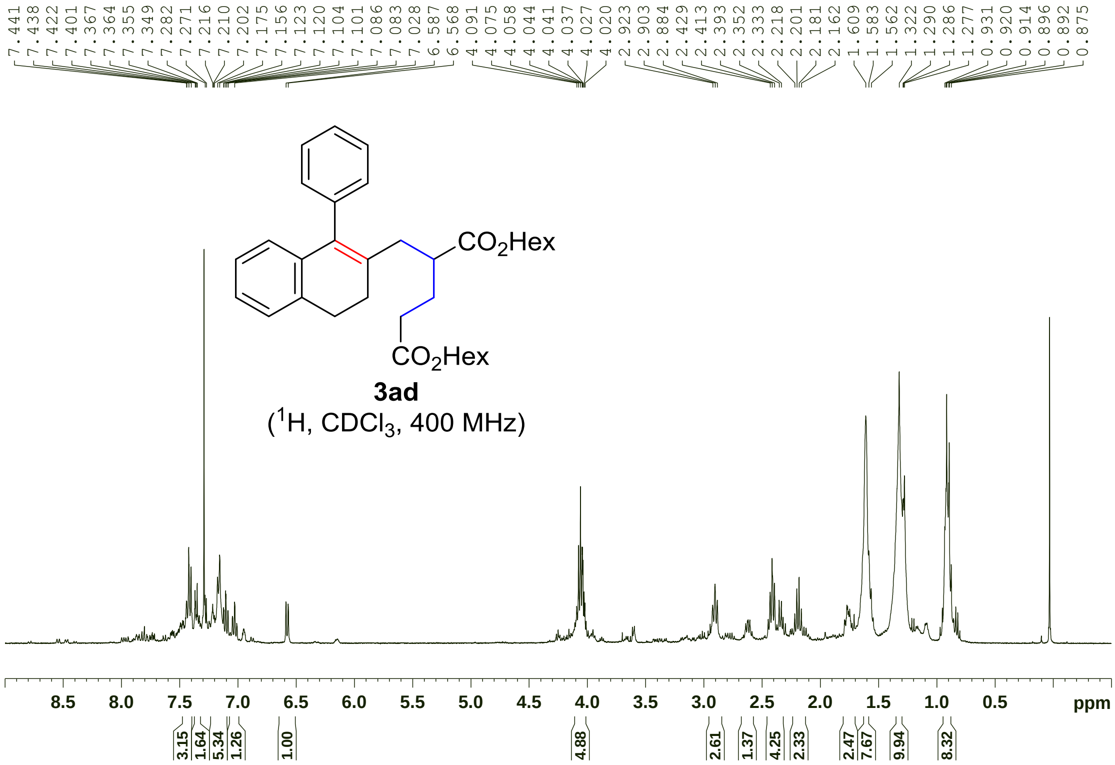
7.441 7.438 7.422 7.401 7.367 7.364 7.355 7.349 7.282 7.271 7.216 7.210 7.202 7.175 7.156 7.123 7.120 7.104 7.101 7.086 7.083 7.028 6.587 6.568 4.091 4.075 4.058 4.044 4.041 4.037 4.027 4.020 2.923 2.903 2.884 2.429 2.413 2.393 2.352 2.333 2.218 2.201 2.181 2.162 1.609 1.583 1.562 1.322 1.290 1.286 1.277 0.931 0.920 0.914 0.896 0.892 0.875
CO2Hex
CO2Hex
3ad
(1H, CDCl3, 400 MHz)
8.5 8.0 7.5 7.0 6.5 6.0 5.5 5.0 4.5 4.0 3.5 3.0 2.5 2.0 1.5 1.0 0.5 ppm
3.15 1.64 5.34 1.26 1.00 4.88 2.61 1.37 4.25 2.33 2.47 7.67 9.94 8.32

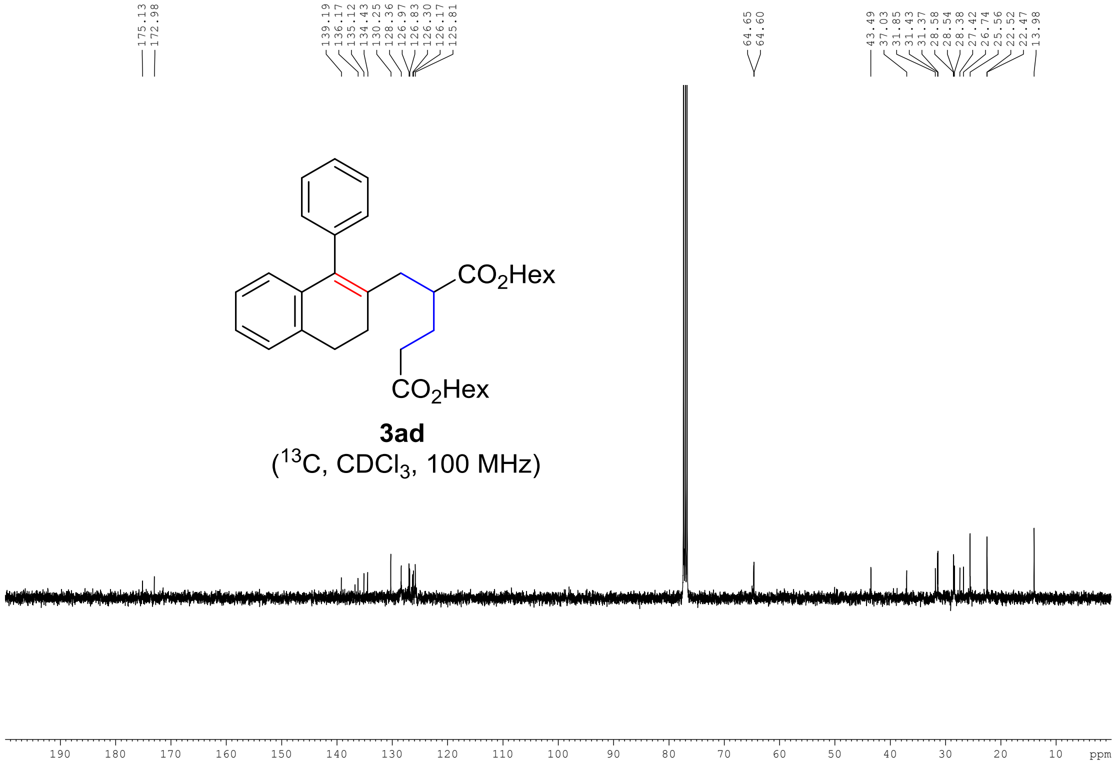
175.13 172.98 139.19 136.17 135.12 134.43 130.25 128.36 126.97 126.83 126.30 126.17 125.81 64.65 64.60 43.49 37.03 31.85 31.43 31.37 28.58 28.54 28.38 27.42 26.74 25.56 22.52 22.47 13.98
CO2Hex
CO2Hex
3ad
(13C, CDCl3, 100 MHz)
190 180 170 160 150 140 130 120 110 100 90 80 70 60 50 40 30 20 10 ppm

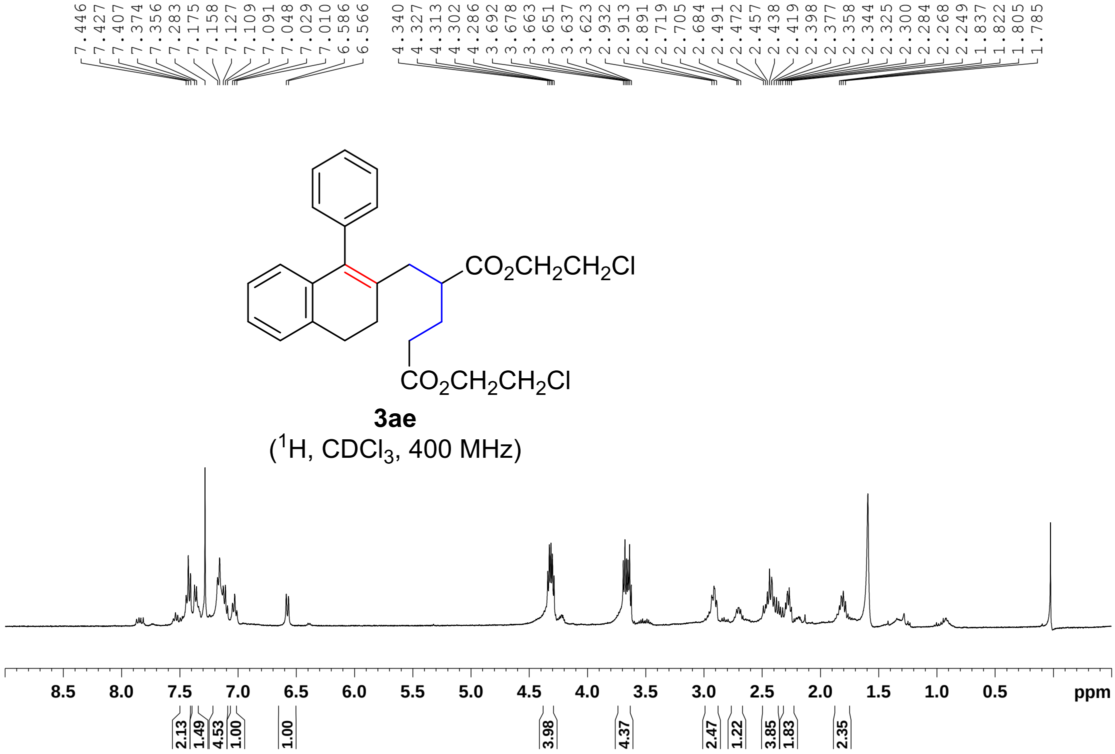
7.446 7.427 7.407 7.374 7.356 7.283 7.175 7.158 7.127 7.109 7.091 7.048 7.029 7.010 6.586 6.566
4.340 4.327 4.313 4.302 4.286 3.692 3.678 3.663 3.651 3.637 3.623 2.932 2.913 2.891 2.719 2.705 2.684 2.491 2.472 2.457 2.438 2.419 2.398 2.377 2.358 2.344 2.325 2.300 2.284 2.268 2.249 1.837 1.822 1.805 1.785
$CO_2CH_2CH_2Cl$
$CO_2CH_2CH_2Cl$
3ae
(^1H, $CDCl_3$, 400 MHz)
8.5 8.0 7.5 7.0 6.5 6.0 5.5 5.0 4.5 4.0 3.5 3.0 2.5 2.0 1.5 1.0 0.5 ppm
2.13 1.49 4.53 1.00 1.00 3.98 4.37 2.47 1.22 3.85 1.83 2.35

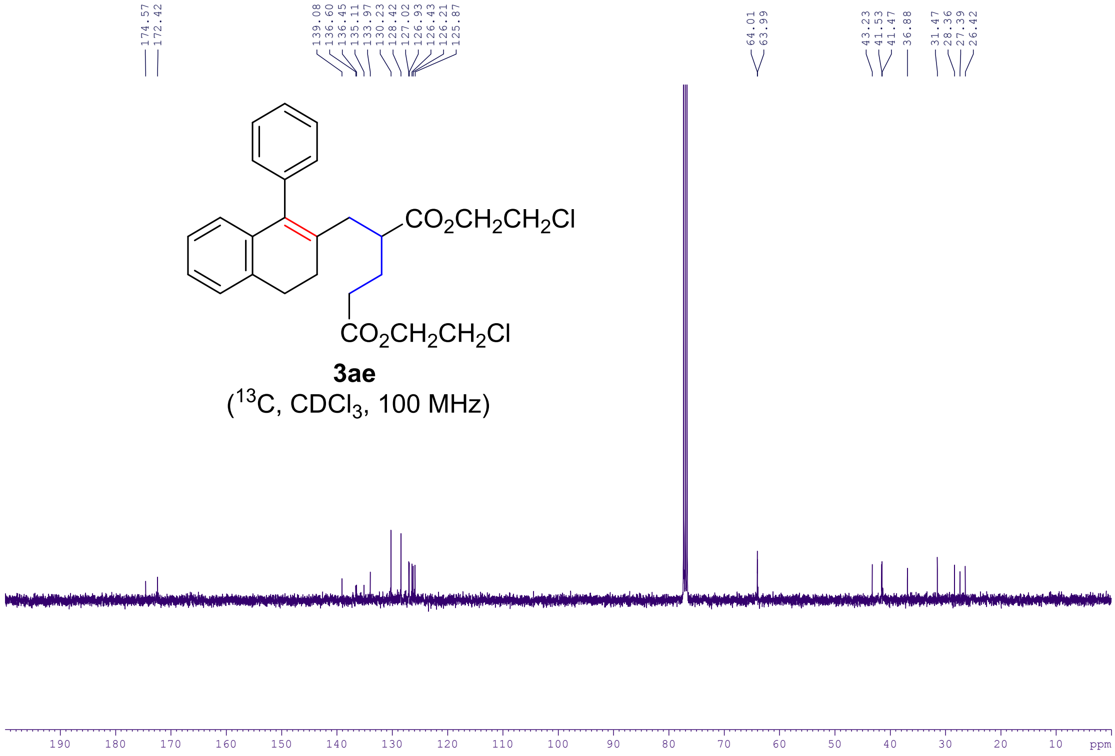
174.57 172.42
139.08 136.60 136.45 135.11 133.97 130.23 128.42 127.02 126.93 126.43 126.21 125.87
64.01 63.99
43.23 41.53 41.47 36.88 31.47 28.36 27.39 26.42
$CO_2CH_2CH_2Cl$
$CO_2CH_2CH_2Cl$
3ae
(^{13}C, $CDCl_3$, 100 MHz)
190 180 170 160 150 140 130 120 110 100 90 80 70 60 50 40 30 20 10 ppm
